# A multimodal evidence-driven framework for clinical decision support in cognitive impairment

**DOI:** 10.1038/s41746-026-03048-9

**Published:** 2026-07-20

**Authors:** Shicong Hu, Xiaoyang Sheng, Feng-ao Wang, Enyuan Zhao, Tianyi Fei, Lu Zhou, Xuguang Zhang, Fangfang Qu, Yixue Li

**Affiliations:** 1 Key Laboratory of Systems Health Science of Zhejiang Province, School of Life Science, Hangzhou Institute for Advanced Study, University of Chinese Academy of Sciences, Hangzhou, China; 2 Guangzhou National Laboratory, Guangzhou, China; 3 School of Life Sciences, Peking University, Beijing, China; 4 School of Life Sciences and Technology, ShanghaiTech University, Shanghai, China; 5 GMU-GIBH Joint School of Life Sciences, Guangdong Provincial Key Laboratory of Protein Modification and Disease, The Guangdong-Hong Kong-Macao Joint Laboratory for Cell Fate Regulation and Diseases, Guangzhou Medical University, Guangzhou, China; 6 Bioland Laboratory, Guangzhou, China; 7 Feihe Reseach Institute, Heilongjiang Feihe Dairy Co., Ltd., Chaoyang District, Beijing, China; 8 School of Life Sciences and Biotechnology, Shanghai Jiao Tong University, Shanghai, China; 9 Shanghai Institute of Nutrition and Health, Chinese Academy of Sciences, Shanghai, China

**Keywords:** Computational biology and bioinformatics, Health care, Medical research

## Abstract

Deep learning approaches for cognitive impairment diagnosis have shown considerable promise, but their clinical translation remains limited by poor interpretability and weak linkage between model outputs and established medical evidence. Here we developed the Multimodal Evidence-Driven Reasoning Framework (MEDRF), which integrates a Multimodal Hierarchical Cascade (mHC) classifier with a retrieval-augmented large language model (RAG-LLM) for evidence-guided reasoning. MEDRF leverages routinely collected non-invasive data from clinical profiles and structural MRI to identify cognitive impairment stages and etiologies. Across 15 diagnostic labels, mHC outperformed flat multimodal baselines, supporting hierarchical diagnostic modeling. When the mHC was evaluated under progressive feature masking, performance declined with increasing missingness, whereas RAG-LLM correction mitigated this effect, especially under severe sparsity. In external validation on a heterogeneous cohort with primary labels, integrating RAG-LLM with the mHC improved all evaluation metrics, increasing overall accuracy from 0.706 ± 0.038 to 0.753 ± 0.032. The RAG module resolves ambiguous predictions by retrieving analogous cases and supporting textual evidence, enabling multi-hop reasoning across conflicting clinical cues. Physician review further indicated favorable quality and perceived usefulness of the generated reports. By synthesizing hierarchical prediction with evidence-grounded reasoning, MEDRF provides a robust interpretable framework for decision support, particularly in incomplete or diagnostically ambiguous presentations.

## Introduction

According to the World Health Organization, cognitive impairment has emerged as a significant public health challenge amidst accelerating population aging, with dementia currently ranking as the seventh leading cause of death globally^1,2^. In detail, the clinical presentation of cognitive impairment is highly heterogeneous, spanning a continuum from normal cognition (NC) to mild cognitive impairment (MCI), and ultimately to dementia (DE)^3^. The National Institute on Aging (NIA) estimates that approximately 5-10% of individuals with MCI progress to full-blown DE annually^4^. Nevertheless, the clinical diagnosis of MCI is exceptionally challenging, as its symptoms often overlap with those of benign senescence or the incipient signs of dementia, creating ambiguous clinical boundaries. This diagnostic uncertainty not only confounds clinicians but also poses a fundamental difficulty for computational models in defining precise decision boundaries^5^. The early and precise subtyping of cognitive impairment is of critical clinical importance. For instance, MCI can be subclassified into amnestic and non-amnestic forms^6,7^. The former is highly predictive of progression to Alzheimer’s disease^8^, whereas the latter may evolve into other dementia types^9,10^, such as vascular dementia or frontotemporal dementia. Dementia itself encompasses a variety of etiologies, including mixed pathologies. Given the significant differences in disease progression trajectories and therapeutic strategies among various subtypes and etiologies^11,12^, accurate classification is an essential precondition for the implementation of personalized medicine^13,14^.

Current clinical diagnostics for cognitive impairment are beset by several problems. Primarily, while advanced biomarkers, including those from cerebrospinal fluid (CSF) tests or Positron Emission Tomography (PET) scans^15^, offer early pathological insights, their highly invasive or costly nature precludes widespread adoption. This challenge is particularly acute in regions with limited medical resources^16,17^, often leading to diagnoses that are time-consuming, labor-intensive, and prone to error, thereby exacerbating the healthcare burden^18^. Furthermore, tools capable of effectively integrating massive, heterogeneous datasets are still lacking^19^. These constraints highlight a critical need to develop novel tools capable of synergizing inexpensive, accessible data with enhanced human-computer interaction^20^. This would not only boost diagnostic speed and precision but also lay a viable groundwork for scalable, precision-based early screening initiatives^21^.

Prevailing machine learning (ML) approaches for diagnosing cognitive impairment have prioritized the optimization of probabilistic outputs, often at the expense of clinical interpretability and external validity^22–24^. Although the advent of explainable AI (XAI) methods like SHapley Additive exPlanations (SHAP) offers a semblance of transparency by quantifying feature contributions to model predictions, these methods fundamentally report statistical correlations, not causation^25,26^. Consequently, they fall short of providing the clinically actionable, evidence-driven causal inferences required for personalized patient diagnosis. Large Language Models (LLMs) ^27,28^ present a novel paradigm by generating narrative reports to articulate diagnostic decisions^29,30^, however, their utility is often compromised by inferior classification accuracy compared to bespoke ML models^31^ and a susceptibility to hallucination or flawed reasoning, rendering their outputs unreliable for the rigorous demands of clinical settings^32,33^. For example, Leng et al. developed a GPT-4o-powered framework to classify CI stages from EHR clinical notes, achieving high accuracy in distinguishing cognitively unimpaired, MCI, and dementia cases^34^. Retrieval-augmented generation (RAG) further bolsters LLM performance in CI decision support by improving factual grounding, transparency, and hallucination mitigation without compromising accuracy^35^. Moreover, models reliant on a single modality fail to capture the multifaceted nature of the disease^36^, in contrast to multimodal models that integrate diverse data sources, such as imaging and clinical assays, which yield higher diagnostic precision and cross-cohort generalizability. At the same time, many multimodal diagnostic models rely on flat prediction structures that do not reflect the hierarchical organization of clinical cognitive diagnosis^37^. In practice, clinicians typically first determine the primary diagnostic state and then refine judgment toward subtype- or etiology-level interpretation. A central challenge, therefore, is to develop a framework that combines multimodal predictive strength with interpretable, evidence-guided reasoning in a form that is aligned with this hierarchical diagnostic process and remains informative in diagnostically ambiguous settings.

Here, we developed the Multimodal Evidence-Driven Reasoning Framework (MEDRF), which combines a Multimodal Hierarchical Cascade (mHC) classifier with a retrieval-augmented large language model (RAG-LLM) for evidence-guided diagnosis across the cognitive spectrum. The mHC is designed to mirror the staged structure of clinical cognitive diagnosis, in which broad cognitive states are first established and then refined into more specific subtypes or etiologies. This formulation is well suited to cognitive disorders, where phenotypic overlap between adjacent categories is substantial, disease-stage boundaries are often ambiguous, and fine-grained classes are markedly imbalanced. Accordingly, the mHC performs sequential coarse-to-fine classification using multimodal non-invasive clinical data, including demographics, medical history, cognitive assessments, and structural MRI (T1- and T2-weighted). Because the primary diagnostic label governs downstream routing, errors at the top level can propagate through the hierarchy and impair subsequent subtype and etiology prediction. MEDRF addresses this vulnerability by using an RAG-LLM to selectively reevaluate uncertainty-sensitive top-level decisions in light of retrieved knowledge and similar cases. When the primary label is corrected, the updated routing prior is propagated to downstream tasks, thereby improving fine-grained discrimination while also enabling traceable, patient-specific reasoning reports. In this way, MEDRF integrates hierarchical multimodal prediction with evidence-guided correction to support accurate and clinically grounded decision-making.

## Results

### Study design and participants

We developed and evaluated the Multimodal Evidence-Driven Reasoning Framework (MEDRF) using three large, publicly available cohorts of individuals spanning the cognitive spectrum. The National Alzheimer’s Coordinating Center^38^ (NACC; *n* = 10,840) and Open Access Series of Imaging Studies 3^39^ (OASIS-3; *n* = 2140) were pooled for stratified five-fold cross-validation, whereas the Alzheimer’s Disease Neuroimaging Initiative^40^ (ADNI; *n* = 706) was reserved as an independent external validation cohort.

To match the granularity of clinical reasoning, we adopted a hierarchical labeling system capturing both cognitive stage and etiologic subtype. At the primary label level, participants were categorized into three clinical states: normal cognition (NC), mild cognitive impairment (MCI), and dementia (DE). To further capture the heterogeneity of the disease, the second label level introduced a finer-grained label for both MCI and DE. MCI was subcategorized into five groups based on the pattern of impaired cognitive domains: amnestic single-domain (MCI_A), amnestic multi-domain (MCI_AM), non-amnestic single-domain (MCI_Na), non-amnestic multi-domain (MCI_NaM), and a preclinical state of cognitive impairment not meeting full MCI criteria (CI). Dementia was further classified into seven etiologic categories based on the primary clinical diagnosis, comprising Alzheimer’s disease (AD), Lewy body dementia (LBD), vascular cognitive impairment (VD), frontotemporal dementia (FTD), movement disorder–associated dementia, reversible or secondary causes (EXC), psychiatric-related cognitive impairment (PSY), and other or unknown etiologies (ODE), yielding 15 distinct labels in total. The distribution and hierarchical co-occurrence of these diagnostic labels are visualized in Supplementary Fig. 1. This granular classification framework established a structured foundation for subsequent multimodal model training and its clinical validation. Baseline demographic and clinical characteristics, including age, sex, education, Clinical Dementia Rating (CDR), and MRI availability, are summarized in Table 1 for each cohort and diagnostic group.

**Table 1:** Patient characteristics at baseline

Dataset (group)	Age, mean ± s.d.	Male, *n* (%)	Education(y), mean ± s.d.	Race (White,Black,Asian, American Indian, Pacific, multirace), *n*	CDR, mean ± s.d.	MRIs available (T1, T2),n
**NACC**						
NC [*n* = 6,361]	70.35 ± 10.29	2185 (34.35%)	16.07 ± 2.89	5,204, 907, 105, 63, 3, 79	0.06 ± 0.16	3,542, 3,072
MCI [*n* = 2,647]	73.14 ± 9.54	1320 (49.87%)	15.48 ± 3.31	2,032, 505, 47, 30, 1, 32	0.44 ± 0.21	1,568, 1,496
DE [*n* = 1,832]	72.69 ± 10.53	940 (51.31%)	15.16 ± 3.34	1,634, 135, 33, 6, 0, 24	1.03 ± 0.61	845, 782
CI [*n* = 302]	71.00 ± 9.50	126 (41.72%)	14.57 ± 3.89	187, 98, 5, 5, 0, 7	0.29 ± 0.26	185, 183
MCI_A [*n* = 827]	74.39 ± 8.73	455 (55.02%)	15.55 ± 3.37	719, 81, 19, 6, 0, 2	0.48 ± 0.17	433, 411
MCI_AM [*n* = 1037]	73.09 ± 9.15	536 (51.69%)	15.76 ± 3.05	769, 223, 17, 11, 0, 17	0.47 ± 0.16	677, 622
MCI_Na [*n* = 284]	73.00 ± 10.60	122 (42.96%)	15.32 ± 3.04	215, 56, 3, 5, 1, 4	0.38 ± 0.28	157, 161
MCI_NaM [*n* = 197]	71.60 ± 12.13	81 (41.12%)	15.37 ± 3.52	142, 47, 3, 3, 0, 2	0.41 ± 0.20	116, 119
AD [*n* = 1500]	73.89 ± 9.81	722 (48.13%)	15.13 ± 3.37	1,324, 122, 29, 6, 0, 19	1.03 ± 0.61	660, 605
LBD [*n* = 169]	74.21 ± 8.16	132 (78.11%)	16.15 ± 3.02	156, 9, 2, 0, 0, 2	1.15 ± 0.65	59, 65
VD [*n* = 192]	78.94 ± 7.90	104 (54.17%)	15.11 ± 3.67	154, 27, 7, 0, 0, 4	1.04 ± 0.63	111, 110
FTD [*n* = 153]	67.00 ± 9.70	88 (57.52%)	15.54 ± 2.79	148, 1, 2, 0, 0, 2	1.01 ± 0.69	94, 88
EXC [*n* = 101]	68.57 ± 14.61	62 (61.39%)	14.52 ± 3.24	88, 12, 1, 0, 0, 0	0.96 ± 0.54	66, 59
PSY [*n* = 59]	70.16 ± 8.87	27 (45.76%)	15.03 ± 3.32	54, 2, 0, 0, 0, 3	1.00 ± 0.54	45, 37
ODE [*n* = 122]	68.81 ± 14.10	76 (62.30%)	14.80 ± 3.44	108, 11, 2, 0, 0, 1	1.07 ± 0.62	52, 44
*P*-value	7.4049e−96	1.9691e−102	1.3039e−51	1.8144e−57	1.0000e−300	
**OASIS**						
NC [*n* = 1,682]	70.30 ± 8.65	694 (41.26%)	16.05 ± 2.53	1,459, 210, 10, 1, 0, 2	0.00 ± 0.02	1,680, 1,621
MCI [*n* = 136]	75.29 ± 6.81	68 (50.00%)	15.48 ± 2.72	124, 12, 0, 0, 0, 0	0.50 ± 0.11	136, 134
DE [*n* = 322]	76.07 ± 7.48	193 (59.94%)	14.98 ± 2.95	277, 43, 1, 0, 0, 1	0.69 ± 0.28	322, 310
CI [*n* = 65]	75.49 ± 7.40	33 (50.77%)	15.12 ± 2.65	60, 5, 0, 0, 0, 0	0.49 ± 0.14	65, 63
MCI_A [*n* = 32]	74.77 ± 6.57	17 (53.12%)	15.59 ± 2.53	29, 3, 0, 0, 0, 0	0.50 ± 0.00	32, 32
MCI_AM [*n* = 35]	75.54 ± 5.98	16 (45.71%)	15.63 ± 2.87	32, 3, 0, 0, 0, 0	0.51 ± 0.08	35, 35
MCI_Na [*n* = 3]	73.70 ± 9.63	1 (33.33%)	18.67 ± 2.31	2, 1, 0, 0, 0, 0	0.50 ± 0.00	3, 3
MCI_NaM [*n* = 1]	74.05	1 (100.00%)	20.00	1, 0, 0, 0, 0, 0	0.50	1, 1
AD [*n* = 77]	75.22 ± 6.75	44 (57.14%)	14.90 ± 2.95	69, 6, 1, 0, 0, 1	0.66 ± 0.27	77, 71
LBD [*n* = 2]	84.98 ± 4.20	2 (100.00%)	19.00 ± 1.41	2, 0, 0, 0, 0, 0	1.50 ± 0.71	2, 1
VD [*n* = 17]	78.04 ± 7.14	10 (58.82%)	14.59 ± 3.28	15, 2, 0, 0, 0, 0	0.71 ± 0.25	17, 17
FTD [*n* = 3]	77.01 ± 3.83	2 (66.67%)	14.67 ± 2.31	3, 0, 0, 0, 0, 0	0.67 ± 0.29	3, 3
EXC [*n* = 19]	78.10 ± 8.24	11 (57.89%)	15.16 ± 2.24	17, 2, 0, 0, 0, 0	0.61 ± 0.21	19, 17
PSY [*n* = 4]	75.99 ± 7.72	2 (50.00%)	14.25 ± 3.30	4, 0, 0, 0, 0, 0	0.50 ± 0.00	4, 4
ODE [*n* = 233]	76.18 ± 7.72	141 (60.52%)	14.94 ± 2.94	199, 34, 0, 0, 0, 0	0.71 ± 0.29	233, 227
*P*-value	7.8599e−51	1.0450e−09	2.1456e−14	N.A.	1.0000e−300	
**ADNI**						
NC [*n* = 254]	73.41 ± 7.22	112 (44.09%)	16.60 ± 2.29	119, 4, 2, 0, 0, 129	0.00 ± 0.00	151,126
MCI [*n* = 218]	73.40 ± 7.82	140 (64.22%)	15.69 ± 2.73	107, 2, 1, 1, 0, 107	0.50 ± 0.08	158,71
DE [*n* = 234]	75.06 ± 7.50	130 (55.56%)	15.82 ± 2.69	109, 5, 3, 0, 0, 117	0.78 ± 0.27	197,18
*P*-value	1.4972e−01	6.0326e−05	9.6890e−04	N.A.	1.6739e−249	

Summary of demographic and clinical attributes for the training and testing cohorts used in the study. Each cohort section details the mean age (with standard deviation), male participant count (with percentage), average years of education (with standard deviation), racial demographics (with counts for each racial category), clinical dementia rating (CDR) breakdown (with counts for each category), and the availability of MRI types (T1, T2). Statistical significance for between-group comparisons was assessed using one-way ANOVA for continuous variables (age, education), and chi-squared tests for categorical variables (gender, race, CDR), with *p*-values reported in scientific notation for each comparison. Statistical comparisons were not concluded for differences in MRI availability. The N.A. entries in the table signify scenarios when statistical tests could not be concluded and when data were not available.

### Interpretable Multimodal Evidence-Driven Framework integrating hierarchical deep learning classification and RAG-LLM

**Fig. 1:**
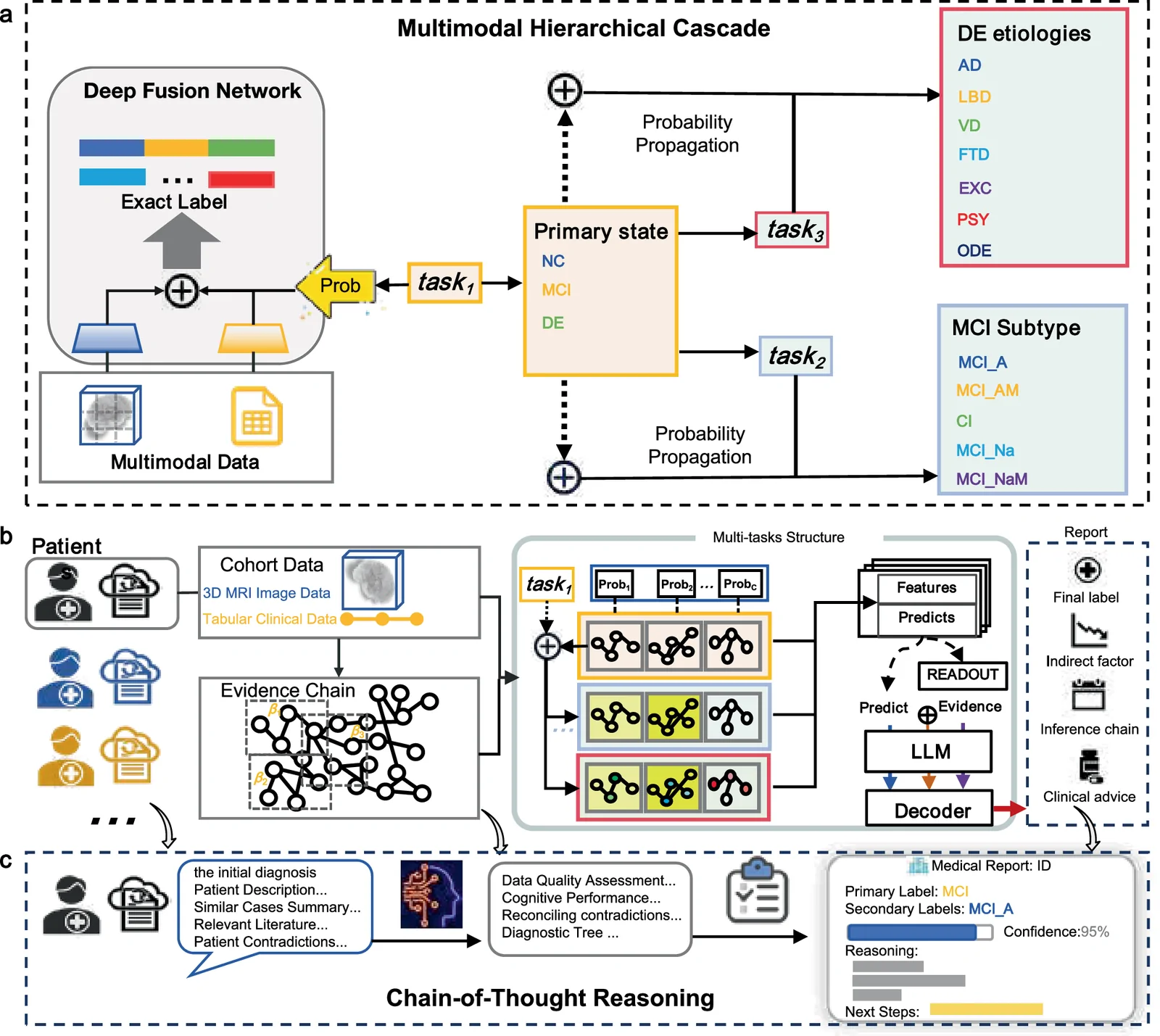
Overview of the Multimodal Evidence-Driven Reasoning Framework (MEDRF). **a** Architecture of the multimodal hierarchical cascade (mHC). Left: The deep fusion network employs dual-stream encoders to fuse 3D MRI and tabular clinical data into a unified latent representation. Right: The Hierarchical mirrors clinical taxonomy, decomposing diagnosis into three progressive tasks: Primary State (Task 1), MCI Subtypes (Task 2), and Dementia Etiologies (Task 3). Probability propagation (dotted arrows) ensures that superordinate predictions explicitly condition and constrain the search space for fine-grained subtyping. **b** End-to-end system workflow. Raw patient data (left) is processed through the trained multi-task structure (center), generating probabilistic predictions, visual features, and an interpretability evidence chain. **c** Mechanism of Chain-of-Thought (CoT) reasoning. The RAG-LLM synthesizes patient profiles with retrieved similar cases and medical guidelines. It executes a multi-step reasoning process—validating data quality, analyzing cognitive performance, and reconciling conflicting evidence—to produce a transparent, verifiable clinical report (right) containing confidence scores and next-step recommendations.

MEDRF is structured as a closed Feature–Evidence–Decision loop comprising two main components (Fig. 1). First, a multimodal hierarchical cascade (mHC) integrates heterogeneous clinical data including demographics, medical history, neuropsychological scores, and T1/T2 structural MRI features through modality-specific encoders and a multi-stage fusion network to generate calibrated probabilities across the 15 diagnostic labels. Its architecture mirrors clinical workflows: an upstream network infers the primary state (NC, MCI, DE), and its posterior probabilities are propagated as prior context to downstream subnetworks that refine MCI subtypes and dementia etiologies.

Second, a retrieval-augmented large language model (RAG-LLM) takes the classifier’s tiered predictions together with structured patient profiles to perform multi-hop retrieval and generate an evidence-grounded refinement of the initial output. To achieve this, we curated two key resources: a domain-specific knowledge base from medical literature and a patient case library indexed by semantic embeddings derived from the training cohort (Methods). Prompted with key patient features and the classifier-derived label, the RAG-LLM iteratively queries both resources, assembling a coherent chain of reasoning capable of integrating concordant evidence and resolving contradictions within the multimodal data. Finally, the RAG-LLM produces a verifiable narrative report and delivers a final, evidence-corroborated diagnosis (Supplementary Notes 1 and 2).

### Superior diagnostic performance through hierarchical multimodal integration

**Fig. 2:**
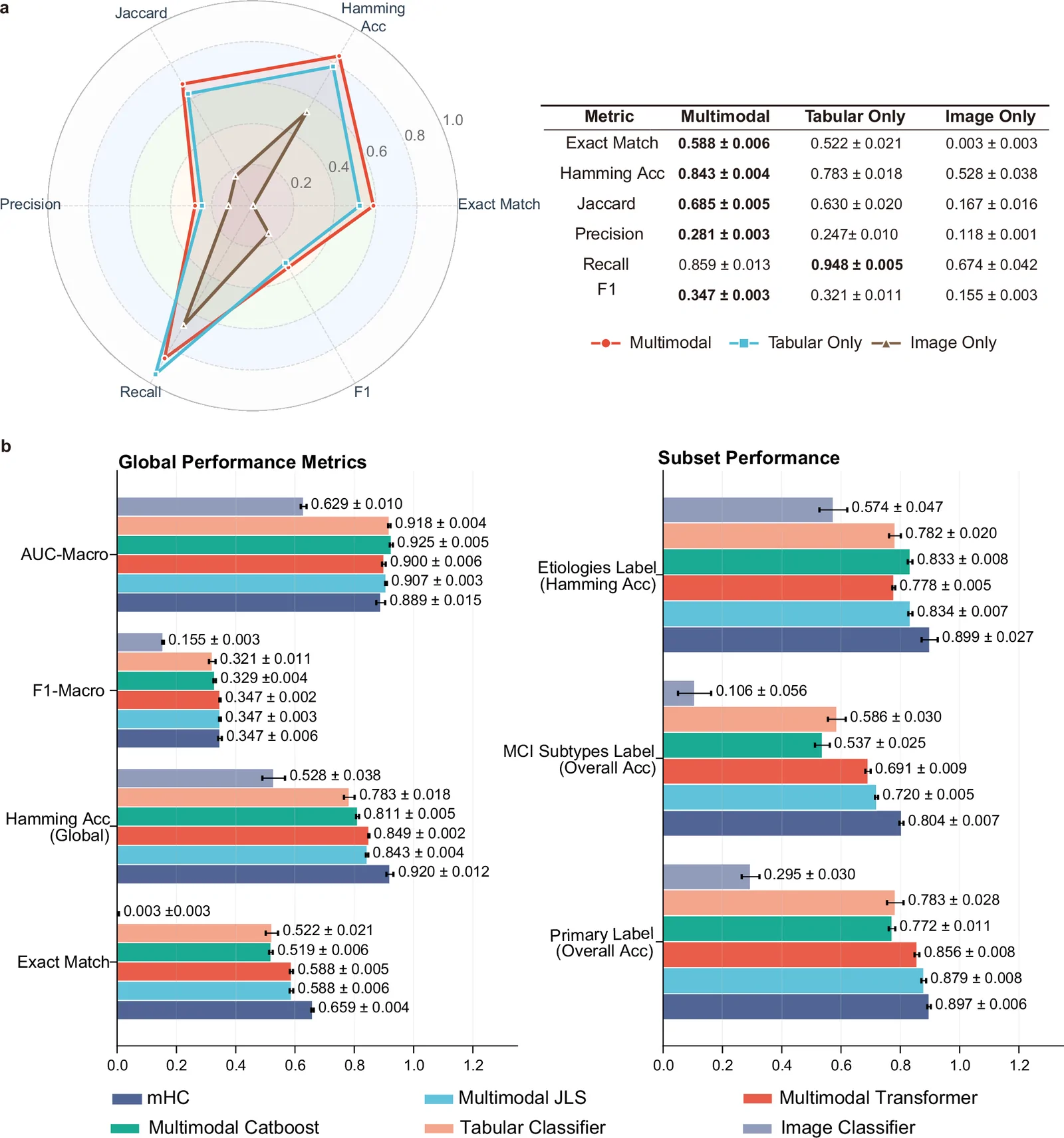
Performance evaluation and ablation study of the hierarchical multimodal diagnostic framework. **a** Radar chart and accompanying table presenting the ablation study results across different input modalities. The performance of the Multimodal Joint Learning Strategy (JLS, red) is compared with Tabular-only (blue) and Image-only (brown) models across six evaluation metrics. Data are presented as the mean (±s.d.). **b** Bar plots comparing the proposed multimodal hierarchical cascade (mHC) with five baseline models: Multimodal JLS, Multimodal Transformer, Multimodal CatBoost, Tabular Classifier, and Image Classifier. The left panel summarizes global performance metrics (Exact Match, Global Hamming Accuracy, Macro-F1, and Macro-AUC), whereas the right panel reports subset-specific performance for Primary Label (overall accuracy), MCI Subtypes Label (overall accuracy), and Etiologies Label (Hamming accuracy). Error bars represent standard deviation.

We first quantified the contribution of each data modality using a multimodal deep learning baseline (Multimodal Joint Learning Strategy, JLS) and its unimodal counterparts (tabular-only and image-only) (Fig. 2a). The multimodal model achieved the highest overall performance, as reflected by an Exact Match score of 0.588 ± 0.006, exceeding both tabular-only (0.522 ± 0.021) and image-only (0.003 ± 0.003) models. These results show that combining structured clinical measures with neuroimaging features provides complementary information that cannot be captured by either modality alone. Complete label-wise performance metrics are provided in Supplementary Table 1.

We next evaluated whether incorporating diagnostic hierarchy could further enhance multimodal classification performance. The mHC framework consists of three specialized subnetworks targeting: (i) primary states (Subset 1: NC, MCI, DE), (ii) MCI subtypes (Subset 2), and (iii) etiologic labels (Subset 3). The posterior probability vector from the primary-state model is concatenated with downstream latent embeddings and used as a soft gating prior during training and inference, enforcing consistency between parent and child labels (Supplementary Fig. 2). Compared with three flat multimodal baselines, including the JLS network, multimodal Transformer, and multimodal CatBoost, the hierarchical design yielded the most robust global performance. mHC achieved a peak Exact Match of 0.659 ± 0.004 and a Hamming Accuracy of 0.920 ± 0.012, substantially surpassing the flat JLS baseline (Exact Match 0.588; Hamming Accuracy 0.843; Fig. 2b, left). Detailed label-wise metrics (Supplementary Table 2) further illustrated the comparative performance of the mHC across the diagnostic hierarchy.

The superiority of the hierarchical approach was particularly evident when analyzing tasks with distinct structural properties (Fig. 2b, right). For the multi-class MCI subtype classification (mutually exclusive categories), the mHC improved accuracy from 0.720 ± 0.005 to 0.804 ± 0.007. This suggests that hierarchical constraints effectively filter irrelevant classes in single-label scenarios. Crucially, for the multi-label etiology classification task (overlapping comorbid conditions), the mHC achieved a Hamming Accuracy of 0.899 ± 0.027 compared with 0.834 ± 0.007 for the baseline. This substantial improvement highlights the model’s ability to capture concurrent etiologies and manage label dependencies without being overwhelmed by the combinatorial complexity that typically challenges flat classifiers. Together, these analyses demonstrate that while multimodal integration provides a strong predictive foundation, explicitly encoding clinical hierarchy further improves both global exact-set agreement and task-specific performance at each granularity level.

To explicitly assess cascading error in the hierarchical framework, we further analyzed confusion matrices at each transition level and compared standard hierarchical inference with a control setting using ground-truth upstream labels (Supplementary Fig. 3). The results showed that misclassifications were primarily concentrated at adjacent diagnostic boundaries, while downstream performance remained relatively stable, indicating limited error propagation through the cascade.

### Generalization assessment of the multimodal hierarchical cascade

We assessed generalizability on the independent ADNI cohort, whose data-collection protocol differs from the training data and exhibits markedly higher feature missingness (approximately 83% – 93% versus mean 48% in NACC/OASIS) together with an overrepresentation of MCI cases, even after excluding visits with > 95% missing features. In this setting, we restricted evaluation to the three primary states (NC, MCI, DE), consistent with label availability in ADNI.

**Fig. 3:**
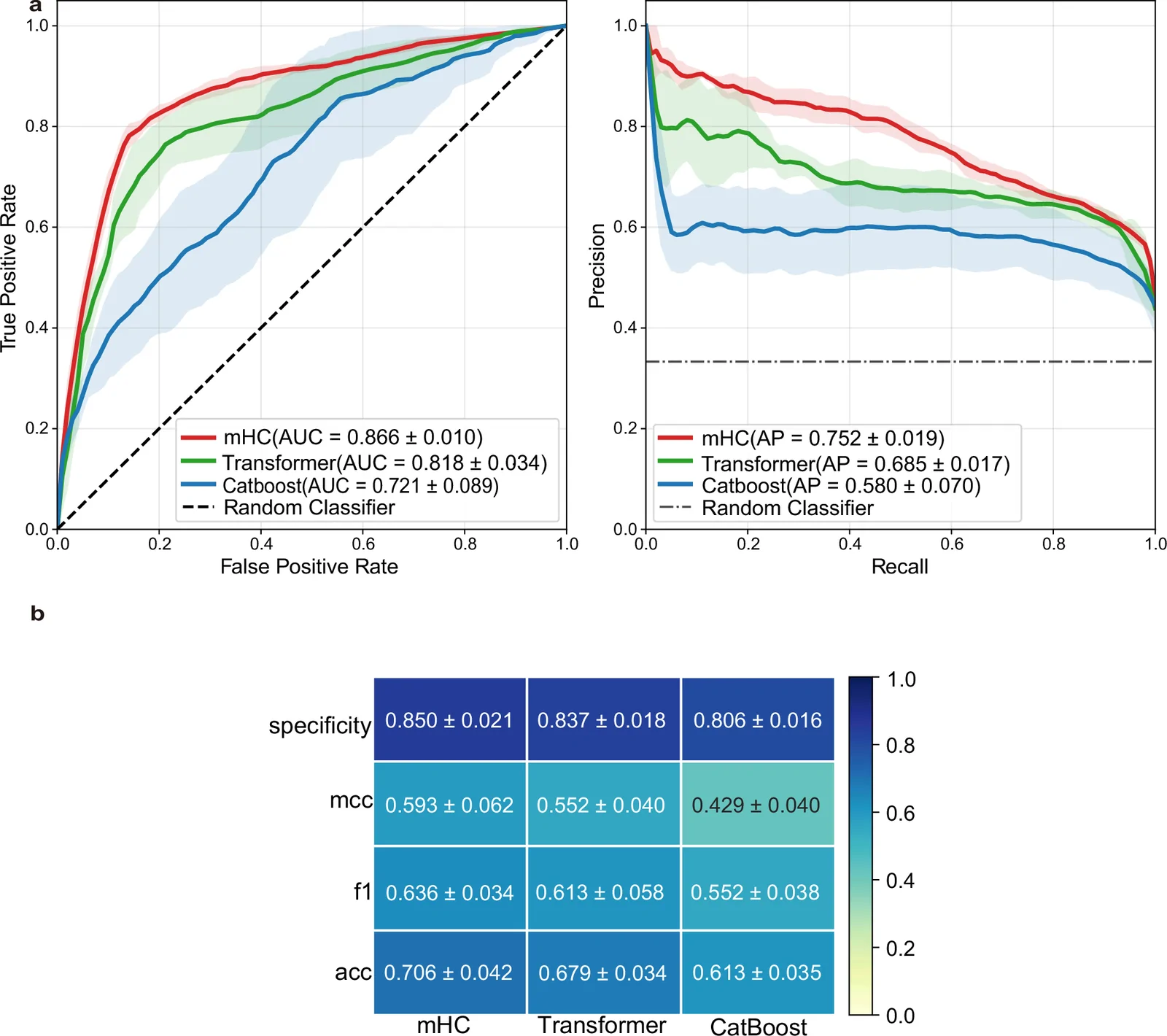
External validation and generalization performance of mHC. **a** Mean ROC and PR curves derived from a five-fold model (mHC vs Transformer vs Catboost). The solid line represents the mean performance, with the shaded band indicating ±1 standard deviation. The dashed line denotes the chance-level baseline. **b** Comparative heatmap of comprehensive performance metrics. Rows display specificity, Matthews correlation coefficient (MCC), F1-score, and accuracy. Cell values denote the mean (±s.d.), with color intensity corresponding to the performance score (0–1 scale). The mHC model consistently outperforms the baselines across all evaluated metrics.

In the external validation cohort, the mHC framework demonstrated superior generalization capabilities compared with the Transformer and CatBoost baseline. Quantitative evaluation confirmed this advantage, with mHC achieving a macro-average AUC of 0.866 ± 0.010 and an average precision (AP) of 0.752 ± 0.019, outperforming the Transformer (AUC: 0.818 ± 0.034; AP: 0.685 ± 0.017) and CatBoost (AUC: 0.721 ± 0.089; AP: 0.580 ± 0.070; Fig. 3a). This performance gain was consistent across all metrics, including a 15.2% relative improvement in accuracy of mHC compared with CatBoost (0.706 vs. 0.679 vs. 0.613 for mHC, Transformer, and CatBoost, respectively) and enhanced specificity (0.850 vs. 0.837 vs. 0.806, Fig. 3b).

However, model uncertainty was higher in borderline samples and in cases with mixed or atypical clinical features, particularly those at diagnostic boundaries where clinical features overlapped between cognitive states. Borderline samples (confidence gap <0.0002) showed 2.56-fold higher prediction variance (coefficient of variation, CV = 0.78 vs. 0.30), indicating reduced stability in diagnostically ambiguous scenarios. These results suggest that boundary ambiguity and multi-etiology presentations remain challenging for the current classifier, thereby motivating the integration of contextual reasoning and case-based evidence via an RAG-LLM component.

### Integrating the RAG-LLM with the mHC enhances external validation performance

To address these limitations, we coupled the mHC with a RAG-LLM framework that leverages both similar cases and curated knowledge to refine predictions in ambiguous situations. For each ADNI test subject, we constructed a structured profile containing baseline clinical variables, radiological findings from UniBrain-derived MRI reports, and the mHC posterior probabilities. Using this profile as a query, the system retrieved the 20 most similar historical cases from a reference library of 12,980 training subjects based on multimodal feature similarity, then synthesized this evidence with classifier predictions using instruction-tuned LLMs. The LLMs were prompted to analyze feature patterns, resolve conflicting indicators, and generate diagnostic reasoning chains that explicitly linked patient characteristics to diagnostic conclusions (Supplementary Fig. 4 and Methods).

**Fig. 4:**
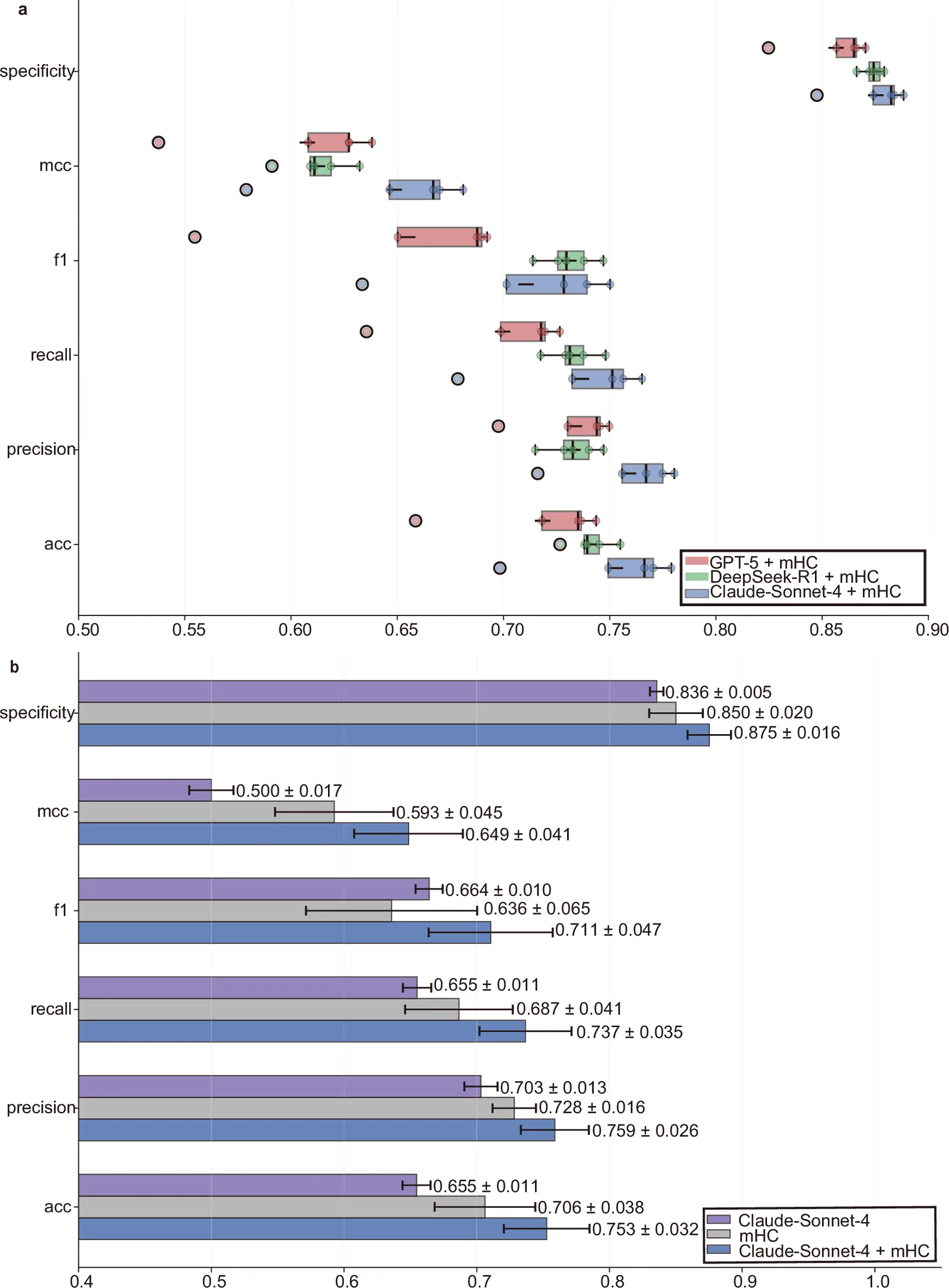
Performance evaluation of MEDRF. **a** Evaluation of model consistency across different foundational RAG-LLMs + mHC. Box plots illustrate the performance distribution across the five fold-derived mHC models for six key metrics (Accuracy, Precision, Recall, F1-score, MCC, and Specificity) using three distinct models: Claude-Sonnet-4, DeepSeek-R1 and GPT-5. **b** Comparative analysis of system architectures. Bar plots display the mean ( ± s.d.) performance across the six metrics for three configurations: Classifier-only, LLM-only, and the proposed LLM + mHC fusion model.

Across the five fold-derived mHC, the MEDRF consistently outperformed the mHC-only on all metrics while evaluation remained confined to the primary label level (Fig. 4). Median accuracy trended highest for Claude-Sonnet-4, with DeepSeek-R1 and GPT-5 close behind; dispersion overlapped across metrics, supporting robustness to provider choice (Fig. 4a). Compared with mHC alone, Claude-Sonnet-4 based MEDRF improved accuracy (0.753 ± 0.032 vs 0.706 ± 0.038), precision (0.759 ± 0.026 vs 0.728 ± 0.016), recall (0.737 ± 0.035 vs 0.687 ± 0.041), F1 (0.711 ± 0.047 vs 0.636 ± 0.065), Matthews correlation coefficient (MCC) (0.649 ± 0.041 vs 0.593 ± 0.045) and specificity (0.875 ± 0.016 vs 0.850 ± 0.020) (Fig. 4b).

Importantly, the LLM alone underperformed both fusion and classifier-only configurations (accuracy 0.655 ± 0.011; F1 0.664 ± 0.010), underscoring complementarity rather than substitutability. This complementarity arises from distinct strengths: the structured classifier excels at pattern recognition from high-dimensional multimodal features, while the LLM leverages clinical reasoning to contextualize ambiguous cases, particularly MCI patients with borderline cognitive scores or conflicting biomarker profiles (Supplementary Fig. 5). Fusion capitalizes on both capabilities, improving MCI F1-score from 0.285 to 0.469 (absolute + 0.184) while maintaining high diagnostic fidelity to the quantitative backbone (Supplementary Table 3). Case-level analysis revealed that LLM-corrected predictions involved patients with missing features, where the LLM successfully integrated fragmented clinical narratives to override classifier predictions (Supplementary Note 3).

### RAG-LLM stabilizes hierarchical diagnosis when feature-derived routing becomes unreliable

To examine the robustness of hierarchical diagnosis under incomplete inputs, we evaluated model performance under progressively increased feature sparsity and compared a flat Multimodal Transformer baseline, the mHC, and the mHC augmented with RAG-LLM correction. Under the standard feature-availability setting used in the main analysis (native missingness ~48%), the mHC showed strong internal performance. We therefore introduced additional masking ratios of 0%, 30%, 60%, and 90%, corresponding to effective overall missingness levels of 52.0%, 63.6%, 79.2%, and 94.8%, respectively (Methods; Supplementary Fig. 6).

At the baseline with a mask ratio of zero, which represents native missingness without additional artificial masking, the mHC showed only a modest advantage over the flat Transformer baseline and did not reach its best performance across the sparsity-stress settings. RAG-LLM correction improved global performance under this condition, increasing F1-macro from 0.287 ± 0.025 to 0.338 ± 0.029, with parallel gains in AUC-macro and AUPR-macro. This improvement was driven mainly by recovery of the upstream diagnostic decision, as primary diagnostic F1 increased from 0.580 ± 0.038 to 0.739 ± 0.045, whereas gains at the subtype and etiology levels were limited. These findings indicate that even without additional artificial masking, native missingness and cohort-specific feature patterns can lead to unstable top-level routing, creating room for RAG-LLM correction.

At moderate sparsity (mask ratio = 0.3), mHC performance was stronger than at the no-additional-masking baseline and remained relatively stable after RAG correction. The mHC achieved an F1-macro of 0.358 ± 0.031 and an AUC-macro of 0.819 ± 0.032, compared with 0.371 ± 0.023 and 0.822 ± 0.022, respectively, after RAG correction. Primary diagnostic F1 remained high and stable (0.767 ± 0.013 to 0.774 ± 0.023), with only minor downstream changes (Supplementary Tables 4 and 5), suggesting that hierarchical routing was relatively stable under this intermediate feature-availability setting. This indicates that model robustness depends not only on the nominal number of available features, but also on whether the retained features provide informative and balanced routing signals.

As sparsity increased to 60%, the hierarchical model became more vulnerable, with mHC performance declining to a level comparable to the flat Transformer. RAG-LLM correction partially offset this degradation, improving F1-macro to 0.322 ± 0.020, largely through recovery at the primary diagnostic stage, where F1 increased from 0.632 ± 0.019 to 0.708 ± 0.025. By contrast, gains on downstream branches were smaller and less consistent, with little improvement in MCI subtype prediction.

Under severe sparsity (mask ratio = 0.9), both the Transformer and the mHC collapsed toward majority-class prediction. Although NC recall approached the ceiling, NC specificity fell to 0.193 ± 0.074 in the Transformer and 0.223 ± 0.023 in the mHC, indicating that both models defaulted to assigning most subjects to NC. In the mHC, this routing failure led to broad performance collapse, with primary diagnostic F1 falling to 0.317 ± 0.019, F1-macro to 0.095 ± 0.010, and AUC-macro to 0.594 ± 0.024 (Supplementary Tables 4 and 5).

RAG-LLM prior correction substantially mitigated this failure mode. At mask ratio = 0.9, NC specificity recovered to 0.800 ± 0.051, and primary diagnostic F1 increased from 0.317 ± 0.019 to 0.608 ± 0.039. Global performance also improved, with F1-macro rising from 0.095 to 0.161, AUC-macro from 0.594 to 0.665, and AUPR-macro from 0.218 to 0.254. In contrast, downstream recovery remained limited, with only small gains in MCI subtype F1 (0.026 ± 0.016 to 0.034 ± 0.017) and etiology F1 (0.049 ± 0.028 to 0.061 ± 0.033).

Overall, the baseline with a mask ratio of zero indicates that greater nominal feature availability does not necessarily translate into better hierarchical diagnosis, as mHC performance under this setting without additional masking was lower than under moderate masking. However, under severe feature sparsity, the hierarchical model showed the most pronounced degradation, consistent with the vulnerability of upstream routing to highly incomplete inputs. The improvement from RAG-LLM correction was therefore not simply proportional to the masking ratio, but was closely tied to the reliability of the uncorrected mHC prediction itself. Larger gains were observed when the original mHC prior was less reliable, including both the baseline and the severe sparsity condition. These findings suggest that RAG-LLM correction primarily acts as a stabilizing prior for fragile top-level routing, thereby improving global performance and partially preserving downstream hierarchical discrimination when feature-derived predictions become unreliable.

### Clinician evaluation confirms quality and clinical reasoning concordance of MEDRF-generated reports

To complement metric-based evaluation, we next assessed the clinical quality of MEDRF-generated narrative reports through physician review. We collected 400 independent clinician assessments using a 5-point Likert instrument spanning five domains: factual accuracy, diagnostic reasoning, content completeness, clarity and professionalism, and perceived clinical utility and safety. The physician-reviewed set comprised 80 cases, with each case independently evaluated by five clinicians (Details of the participating clinicians are provided in Supplementary Table 7). For descriptive purposes, these cases covered four representative scenarios defined by the relationship among the mHC prediction, the diagnosis expressed in the generated report, and the reference diagnosis, including cases in which both the mHC prediction and the report diagnosis were correct, cases in which the mHC prediction was incorrect but the report diagnosis supported the reference diagnosis, cases in which the mHC prediction was correct but the report diagnosis was incorrect, and cases in which both were incorrect. The evaluation framework showed excellent overall internal consistency, with a Cronbach’s α of 0.951 (95% CI, 0.944 – 0.958). Reliability remained high across all individual domains, with Cronbach’s α values ranging from 0.803 to 0.887, indicating stable physician scoring across report-quality dimensions.

Across all five domains, clinician ratings were uniformly favorable, with mean scores exceeding 4.0 out of 5 (Supplementary Fig. 7). The overall report-quality score was 4.19 ± 0.53. Among individual domains, clarity and professionalism received the highest rating (4.26 ± 0.57), followed by factual accuracy (4.21 ± 0.56), content completeness (4.20 ± 0.60), diagnostic reasoning (4.16 ± 0.61), and perceived clinical utility and safety (4.08 ± 0.67). Median scores for all domains were 4.0, with narrow interquartile ranges, indicating that favorable evaluations were broadly consistent rather than driven by a small subset of highly rated reports. In addition, in 339 of 400 assessments (84.75%), reviewing clinicians judged that the diagnostic impression conveyed by the generated report was concordant with their own preliminary judgment based on the report content. This item was designed to capture clinician-perceived agreement with the report’s diagnostic logic, rather than correctness relative to the reference diagnosis or the underlying model prediction.

Qualitative feedback further clarified both the strengths and the residual limitations of the generated reports (Verbatim comments from participating clinicians are provided in Supplementary Table 8). Physicians most frequently highlighted clear language, logical organization, and relatively complete evidence chains with limited over-interpretation. Recurring concerns included overly generalized or repetitive treatment recommendations, insufficient emphasis on key MRI findings or urgent radiological priorities, occasional statements not fully traceable to the source record, incomplete incorporation of the chief complaint or disease course into the reasoning process, and possible misinterpretation of cognitive scales such as MoCA versus MMSE. Together, these findings indicate that MEDRF-generated reports are broadly acceptable to clinicians and clinically coherent, while also identifying concrete opportunities for refinement in treatment nuance, source traceability, and safety-oriented reporting.

## Discussion

Our findings address three persistent barriers to the clinical deployment of AI for cognitive diagnosis: vulnerability to class imbalance, limited interpretability of deep learning predictions, and reduced robustness across heterogeneous clinical settings. To address these issues, we developed an evidence-driven reasoning framework that combines hierarchical multimodal prediction with retrieval-augmented large language modeling. Within this framework, structured model outputs are coupled to literature-grounded evidence retrieval, enabling diagnostic predictions to be contextualized by traceable supporting information.

The hierarchical design was intended to improve performance under severe sample imbalance by decomposing the diagnostic task into clinically structured stages, each optimized with task-appropriate evaluation priorities. In parallel, the RAG-LLM component provided an interpretable evidence layer that linked model predictions to individualized explanatory reports and source citations. Rather than functioning as an autonomous decision-maker, this component was designed to support clinician auditability by exposing the evidentiary basis and logical consistency of model outputs.

An additional strength of this framework is that its external knowledge layer can be updated independently of the predictive backbone, providing a practical mechanism for incorporating evolving medical evidence and cohort-specific reference information. In our implementation, this knowledge-guided component was used to refine the upstream diagnostic prior under sparse-input conditions, thereby mitigating hierarchical error propagation. Together, these results suggest that combining data-driven prediction with evidence-linked reasoning may offer a practical route toward more transparent and clinically adaptable AI systems for cognitive impairment assessment.

External validation further showed sustained discriminative performance despite heterogeneity in acquisition protocols, cognitive assessments, and cohort composition. This cross-site resilience likely arises from the joint contribution of three factors: the intrinsic stability of the multi-stage, multi-modal architecture, the RAG-LLM’s capacity to enrich sparse patient profiles through knowledge-based inference, and the evidence-guided label correction process. This pattern also helps explain the discrepancy between the image-only and fused models. In our hierarchical multi-label setting, MRI-only prediction is constrained by the high dimensionality of 3D imaging data, limited sample size, and substantial inter-subject variability. By contrast, multimodal fusion can rely on structured clinical variables as a stable anchor while incorporating MRI-derived signals when they provide complementary information, thereby yielding a net gain in performance.

Beyond predictive gains, physician review further supported the clinical quality of the evidence-grounded reports generated by MEDRF. Clinicians rated the reports favorably across factual accuracy, diagnostic reasoning, content completeness, clarity and professionalism, and perceived clinical utility and safety, and the report conclusions showed high concordance with physicians’ preliminary judgments. These findings are consistent with the intended role of the RAG-LLM layer: not to replace clinical decision-making, but to translate structured model outputs into clinically legible narratives whose reasoning can be examined against patient-specific evidence. Qualitative feedback further suggested that the main residual weaknesses lay not in overall coherence, but in treatment specificity, source traceability, and the prioritization of key neuroimaging findings.

Despite its promise for advancing evidence-driven diagnostics, our framework has limitations. First, performance on certain challenging diagnostic labels could be improved, due to the intrinsic heterogeneity of cognitive disorders, inconsistencies in diagnostic criteria across sources, and dataset size constraints. Although synthetic augmentation may partially alleviate imbalance, future gains will likely depend more on larger real-world datasets with harmonized annotation and broader representation of atypical cases. In addition, the retrieval module is limited to established medical literature, which may introduce uncertainty when applying generalized knowledge to rare etiologies or patient-specific nuances, weakening evidentiary support. Future iterations should incorporate a dynamic, expert-curated workflow to integrate emerging research. Finally, although external validation demonstrated improved predictive performance and retrospective physician review supported the credibility, readability, and reasoning concordance of the generated reports, their practical usability was not formally evaluated in prospective clinical workflows. Such studies will be necessary to determine whether these reports improve clinician trust, efficiency, or decision quality in routine care.

In summary, this study presents a novel framework that fuses multi-stage, multi-modal classification with evidence-grounded reasoning, paving the way for precise diagnosis of cognitive disorders. By generating evidence-linked reports that were diagnostically informative, it improves not only predictive performance but also the clinical interpretability and trustworthiness of AI-assisted diagnosis. We have rigorously validated this hybrid strategy, blending data-driven insights with knowledge-based rigor. This innovation bridges the gap between AI and clinical practice, offering a scalable paradigm for intelligent diagnostics in complex diseases, although prospective real-world studies will be required to determine its effect on clinical decision-making and patient outcomes.

## Methods

### Participant inclusion and exclusion criteria

The research data used in this study were obtained from NACC^38^ (https://naccdata.org/), OASIS-3^39^ (https://sites.wustl.edu/oasisbrains/), and ADNI^40^ (https://adni.loni.usc.edu/) datasets. The study was conducted in accordance with the Declaration of Helsinki. The NACC data were collected under protocols approved by institutional review boards at each participating Alzheimer’s Disease Research Center; OASIS-3 data were collected under protocols approved by the Institutional Review Board of Washington University; the ADNI was approved by institutional review boards at each participating site. Written informed consent was obtained from all participants in accordance with institutional requirements. All data used in the present study were de-identified and made available for scientific research. As this study involved only secondary analysis of existing de-identified data, with no recruitment of new participants and no study-related intervention, additional ethical approval was not required in accordance with institutional policies and applicable regulations.

Participants were included if they had a definitive clinical diagnosis and at least one valid neuropsychological assessment. All clinical features were harmonized to the Uniform Data Set (UDS) version 3 specification. To assemble the multi-modal dataset, we matched data points by participant ID, enforcing a maximum interval of six months between the clinical assessment and the MRI acquisition date. Priority was given to participants with a complete set of T1-weighted and T2-weighted MRI scans to ensure a comprehensive imaging profile. The final dataset aggregated heterogeneous information across demographics, medical history, neuropsychological batteries, and MRI imaging.

### MRI processing

For the MRI data preprocessing, raw MRI images were registered to the MNI152 standard space using the FLIRT tool^41^ within the FSL software^42^. This initial registration utilized an affine transformation with 12 degrees of freedom to account for inter-subject variations in brain volume and head positioning. Brain extraction was then performed using the Brain Extraction Tool^43^. Skull-stripped images were subsequently non-linearly registered to the MNI152 template, using FNIRT to improve alignment of brain parenchyma. Images were denoised with a 3D Gaussian filter (kernel size 5×5×5, σ = 1.0) and intensity-normalized by scaling voxel values to the [0, 1] range, based on the 1st and 99th percentiles of the brain-masked intensity distribution, to reduce scanner-related variability. Both T1-weighted and T2-weighted images were independently registered to MNI152 standard space using FLIRT with 12 degrees of freedom. After preprocessing, the T2-weighted volumes were resampled to match the T1-weighted voxel grid using trilinear interpolation (scipy.ndimage.zoom, order=1). The co-registered T1 and T2 volumes were concatenated along a fourth dimension to form a two-channel 4D tensor, and subsequently downsampled by a factor of 2 (scale_factor = 0.5) via trilinear interpolation, yielding a final matrix size of 45 × 54 × 45 (approximately 4 mm isotropic resolution).

### Multimodal feature embeddings

Our classifier integrates multimodal inputs, including demographics, clinical history, neuropsychological scores, and structural MRI (T1/T2), to maximize predictive information. To effectively extract and align features from these heterogeneous data types, we designed three specialized encoders that project each modality into a shared, comparable embedding space.

For structural MRI^44^, we developed a lightweight 3D Convolutional Neural Network (3D-CNN) consisting of four convolutional blocks^45^. Channel depth progressively increases while spatial resolution decreases, enabling efficient yet powerful feature extraction with low computational cost. To enhance robustness and generalization, we incorporated stochastic 3D channel dropout. Each convolutional layer is followed by batch normalization and ReLU activation for stable and accelerated training. The network concludes with global average pooling, yielding a 256-dimensional embedding.

Tabular data, including categorical and numerical features, were processed by two separate multi-layer perceptrons (MLPs). For each tabular input, missing entries were first identified during preprocessing and encoded as a numerical binary mask (${\rm{m}}\in {\{0,1\}}^{{\rm{d}}}$), where (${{\rm{m}}}_{{\rm{j}}}=0$) indicates an observed feature and (${{\rm{m}}}_{{\rm{j}}}=1$) indicates a missing or masked feature. The input to each MLP was formed by concatenating (1) the numerically preprocessed feature vector, (2) the binary missingness mask, and (3) random noise substituted at masked positions. This tripartite design allows the model to treat missingness as an informative signal while ensuring that all inputs to the MLPs are valid numerical values. Both MLPs are two-layer networks with ReLU activations and dropout, producing independent 128-dimensional embeddings for categorical and numerical features, respectively.

By design, all encoders produce embeddings of comparable scale, preventing modality dominance due to variance in raw feature magnitudes. The full model is trained end-to-end using joint optimization, enabling all encoders to co-adapt and learn synergistic, cross-modal representations that enhance overall performance.

### Multimodal Hierarchical Cascade (mHC) Architecture

We developed a multi-stage fusion network that hierarchically integrates 3D MRI features with categorical and numerical tabular data^46^, conditioned on diagnostic priors from superordinate classifiers (Supplementary Fig. 2). Four input streams, including 3D-CNN embeddings (256-dim), categorical embeddings (128-dim), numerical embeddings (128-dim), and posterior probabilities ${{\rm{p}}}_{{\rm{prob}}}\in [0,1]$ from the upstream model. The three feature embeddings are concatenated into a 512-dimensional vector (early fusion), while the posterior probabilities are reserved for FiLM conditioning and late fusion.

An MLP block (Linear → BatchNorm → ReLU → Dropout, p = 0.5) reduces the fused vector to 256 dimensions, capturing initial cross-modal interactions. The diagnostic prior is then injected via Feature-wise Linear Modulation (FiLM): a two-layer shared network (15 → 64 → 64, GELU activations) transforms the prior probabilities, and two separate linear projections produce scale (γ, 64 → 256) and shift (β, 64 → 256) parameters that affine-transform the fused representation through a residual connection (x ← x + γ ⊙ x + β). This mimics clinical hierarchical reasoning by allowing higher-level diagnostic context to gate lower-level feature processing.

The modulated features pass through a Squeeze-and-Excitation (SE) block with reduction ratio r = 4 (256 → 64 → 256; GELU, Sigmoid), performing channel-wise recalibration via global average pooling to adaptively emphasize informative channels. A second MLP compresses the representation to 64 dimensions. Late fusion re-incorporates the diagnostic prior by concatenation (64 + 15 = 79), followed by an MLP (79 → 64) and a linear classification head (64 → 15) with learnable temperature scaling (initialized at 2.0) and sigmoid activation for calibrated probabilities.

Training employs random modality dropout with NaN-imputation for missing data, plus residual connections and batch normalization throughout. This architecture combines early/late fusion, FiLM conditioning, and SE attention, which enables robust integration of multimodal signals and diagnostic context.

### Training and evaluation of the multimodal hierarchical cascade

We implemented a stratified five-fold cross-validation framework on the combined NACC and OASIS-3 datasets, partitioning data (80% training, 20% internal test) to preserve the joint distribution of the 15 diagnostic labels. Crucially, the ADNI cohort was reserved exclusively as an independent external test set, ensuring it remained unseen during both model training and hyperparameter optimization.

We designed a multimodal hierarchical cascade (mHC) framework composed of three specialized sub-networks, each targeting a specific diagnostic granularity: Primary States (Subset 1), MCI Subtypes (Subset 2), and Etiologies (Subset 3).

The output probability vector from the Subset 1 model is concatenated with the latent feature embeddings of the Subset 2 and Subset 3 models. This allows the downstream networks to explicitly condition their predictions on the primary diagnostic state. To enforce clinical consistency and leverage hierarchical dependencies, we implemented a probabilistic propagation strategy. The Primary State Model (Subset 1) first computes the probability vector for the coarse-grained classes. These probabilities are then forwarded to the downstream models, serving as soft gating factors during both training and inference. Specifically, the predicted probability of a parent class (e.g., MCI) from the Subset 1 model is used to weight the loss and predictions of the corresponding child classes (e.g., Amnestic MCI) in the Subset 2 model. This ensures that the fine-grained models focus their learning on samples where the primary diagnosis is confident.

Each sub-network was trained to minimize a Class-Balanced Binary Cross-Entropy (BCE) Loss. To mitigate the bias toward majority classes and ensure sensitivity to rare etiologies, we calculated positive class weights (${w}_{c}\,$) based on the inverse class frequency:

1 $${L}_{{total}}=-\frac{1}{N}\sum _{i=1}^{{\rm{N}}}\sum _{c=1}^{C}{w}_{c}[{y}_{i,c}{log}({\hat{y}}_{i,c})+(1-{y}_{i,c}){log}(1-{\hat{y}}_{i,c})]$$

where N is the batch size (*n* = 128 for the primary model; *n* = 256 for the downstream classifiers), C is the number of labels, ${y}_{i,c}$ is the ground truth, and ${\hat{y}}_{i,c}$ is the predicted probability.

Optimization was performed using the AdamW optimizer with an initial learning rate of $1\times {10}^{-4}$ and a weight decay of $1\times {10}^{-3}$. We implemented a dynamic learning rate scheduler (ReduceLROnPlateau), which decayed the learning rate by a factor of 0.5 if the validation loss did not improve for 5 consecutive epochs, with a minimum floor of $1\times {10}^{-6}$. To prevent overfitting, we employed Early Stopping with a patience of 30 epochs, monitoring the macro-averaged F1 score on the validation set. The maximum training duration was set to 300 epochs per fold. At inference, the optimal binary classification threshold for each label was determined by maximizing the F1 score on the validation set.

Unlike standard multi-class classification, our framework requires simultaneous precision across multiple diagnostic granularities. We employed a comprehensive suite of metrics to evaluate performance: Exact Match (Subset Accuracy), defined as the percentage of samples where the predicted label set exactly matches the ground truth vector across all 15 categories. Hamming Loss measures the fraction of incorrect labels to the total number of labels, quantifying the distance between predicted and true label vectors. We reported Macro-F1, Macro-Precision, Macro-Recall, and Macro-AUC to treat all diagnostic classes equally regardless of their prevalence.

### Multi-hop evidence retrieval engine and knowledge base construction

To construct a medical knowledge base for grounding LLM reasoning, we established a systematic pipeline for literature acquisition, preprocessing, indexing, and retrieval. Relevant publications related to alzheimer’s disease and cognitive impairment were retrieved from PubMed, Scopus, and Web of Science using database-specific Boolean search strategies. The search covered publications from January 2000 to December 2024. After record merging and deduplication, 54,003 articles were retained for downstream processing. Non-English publications, retracted papers, records with missing abstracts, and documents with unparseable PDFs were excluded during preprocessing. To improve evidence quality while preserving coverage, articles published after 2017 with more than 10 citations were designated as a high-quality subset and assigned boosted retrieval priority, whereas the remaining eligible records were retained as general references (Supplementary Table 6).

Retrieved PDFs were parsed using GROBID into structured TEI-XML format, cleaned using targeted regular expressions, and converted into plain text while preserving article section hierarchy. Instead of indexing the full article as a single unit, we segmented each document into section-level chunks (e.g., Abstract, Introduction, Methods, Results, and Discussion/Conclusion), and each chunk was indexed as an independent retrieval unit with links to article-level metadata, including title, authors, year, DOI, and citation count. This section-level strategy was adopted to improve retrieval precision for patient-specific clinical queries. Sparse retrieval was implemented using Okapi BM25^47^ in Elasticsearch, with standard English tokenization, stemming, and stop-word removal.

Following MRI preprocessing, the prepared 4D tensors were input into UniBrain^48^ to generate structured radiological reports. This module extracted high-dimensional anatomical features (e.g., cortical atrophy, white matter hyperintensities) from key regions including the hippocampus and entorhinal cortex, mapping visual patterns to standardized radiological terminology for downstream retrieval tasks.

For each patient, baseline clinical variables, structured radiological findings from T1/T2 MRI, and probabilistic predictions from the mHC were aggregated into a unified JSON patient profile. This profile served as the query for retrieval. Evidence retrieval was implemented using the Okapi BM25 algorithm, a probabilistic ranking function that estimates the relevance of documents based on the occurrence of query terms. Unlike simple frequency-based metrics, BM25 incorporates term saturation limits and document length normalization, ensuring that the retrieval process prioritizes specific clinical keywords while mitigating the bias from repetitive terms or varying document lengths. For complex queries requiring multi-hop reasoning (e.g., mechanistic explanations or resolution of conflicting features), the system iteratively refined queries and identified and retrieved salient evidentiary passages from the literature capable of substantiating or contextualizing the model’s prediction.

Finally, the system synthesizes a consolidated, patient-specific report comprising four key components: (i) baseline clinical summary; (ii) critical MRI radiological findings; (iii) calibrated diagnostic probability from the mHC; and (iv) extractive summaries of the most salient supporting literature passages with direct citations.

### RAG framework for resolving diagnostic contradictions

We implemented a retrieval-augmented generation large language model (RAG-LLM) framework to generate verifiable diagnostic reports and explicitly address contradictory multimodal evidence (Supplementary Fig. 4). Multimodal patient features were first translated into natural-language clinical statements and organized into structured diagnostic elements.

Evidence was retrieved through two complementary branches. In the case-based retrieval branch, Sentence-BERT embeddings were used to encode the target patient and a reference cohort (*n* = 12,980). Cosine similarity was computed between the target and all reference cases, and the top 20 most similar historical patients were retrieved together with similarity scores. In the literature retrieval branch, the structured patient profile was submitted to the two-stage BM25 retrieval pipeline described above. The first stage targeted literature relevant to the patient’s core phenotype, whereas the second stage expanded retrieval to atypical, mixed, or internally inconsistent presentations. Retrieved text chunks were merged, deduplicated, and re-ranked before the final passages were passed to the language model.

Outputs from these retrieval branches were routed to two downstream modules: Case Pattern Analysis, which summarized recurrent patterns and divergences among similar historical patients, and Evidence Synthesis, which consolidated literature-supported findings into diagnosis-relevant statements. After structural alignment, diagnostic inclinations, supporting evidence, and exclusion rationales were explicitly linked to traceable sources. These components were then integrated by the Report Generation module into a final diagnostic report with an explicit evidence chain. The prompts and retrieval configurations underlying the RAG-LLM reasoning module are detailed in Supplementary Notes 1 and 2.

By enforcing statement-evidence alignment, the framework was intended to reduce hallucination risk and improve interpretability. In cases with contradictory clinical signs, the evidence was organized into a tripartite structure of conflicting statement–explanatory hypothesis–supporting evidence, enabling clinically interpretable localization of diagnostic disagreement.

### Feature sparsity simulation and RAG-LLM hierarchical correction

To evaluate the robustness of MEDRF under incomplete clinical records, we performed a controlled feature sparsity analysis on the held-out test data from each fold of the five-fold cross-validation. In each fold, 50% of the test subjects were selected to form a sparsity-stress cohort. This selection was performed within each primary diagnostic stratum (NC, MCI, and DE) so that the class distribution of the primary diagnostic categories was preserved. Within each stratum, subjects were ranked by the number of available, non-missing clinical features, and the top 50% with the highest feature availability were selected. This strategy generated a fixed, label-balanced sparsity-stress cohort with maximal baseline feature content, reducing the computational burden of repeated RAG-based inference under multiple sparsity settings, while ensuring that all perturbation experiments were conducted strictly within the held-out test partition and remained isolated from model training and validation procedures.

For each subject in the sparsity-stress cohort, model performance was evaluated under additional masking ratios of 0, 0.3, 0.6, and 0.9 applied to the tabular features. The mask ratio = 0 setting served as the no-additional-masking baseline and therefore retained the native missingness pattern of the selected test subjects. The 0.3, 0.6, and 0.9 settings introduced progressively higher levels of artificial feature sparsity on top of native missingness. Masking was applied only at inference time, and once sparsification was introduced, the original unmasked records were not used for prediction under that masking condition. After feature alignment, masked tabular entries were represented as NaN values during inference. This design provided a controlled robustness stress test to quantify the sensitivity of the hierarchical framework to increasing feature incompleteness while keeping the evaluated subject cohort and label distribution fixed across sparsity levels.

Inference proceeded in a hierarchical manner. First, a multimodal global model generated a 15-dimensional prediction vector, including probabilities for the primary diagnostic categories (NC, MCI, DE), dementia etiologies, and MCI subtypes. The first three dimensions, denoted ${{\rm{P}}}_{{\rm{global}}}$, were used as the primary routing signal in the hierarchical cascade. The remaining downstream tasks were then evaluated using task-specific models conditioned on the multimodal inputs and a prior vector derived from the global prediction. Because downstream branch selection depended on the upstream global state, perturbation of the global prediction under feature sparsity could affect subsequent subtype and etiology inference.

To test whether external knowledge could mitigate sparsity-induced routing degradation, we incorporated a retrieval-augmented generation module as a global-prior correction mechanism. The multimodal global model first produced a 15-dimensional sigmoid-derived prediction vector. Its first three dimensions, denoted (${{\rm{p}}}_{{\rm{global}}}^{{\rm{MM}}}\in \,{[\mathrm{0,1}]}^{3}$), corresponded to the primary diagnostic categories NC, MCI, and DE and were used as the upstream routing prior. Because these scores were generated by sigmoid activation, they were interpreted as independent per-class scores rather than as a normalized softmax distribution.

When RAG outputs for the three primary diagnostic classes were available, the RAG module provided a one-hot auxiliary prior ($\,{r}_{{\rm{global}}}^{{\rm{RAG}}}\in \,{\{\mathrm{0,1}\}}^{3}$) over NC, MCI, and DE. RAG-based correction was applied only when the RAG-predicted class disagreed with the argmax class of (${p}_{{\rm{global}}}^{{\rm{MM}}}$). For such disagreement cases, the corrected global prior was computed by soft interpolation:

2 $${\widetilde{p}}_{{\rm{global}}}=(1-\alpha ){p}_{{\rm{global}}}^{{\rm{MM}}}+\alpha {r}_{{\rm{global}}}^{{\rm{RAG}}}$$

where ($\alpha \,=\,0.65$). If the RAG and multimodal predictions were concordant, or if RAG outputs were unavailable, the original (${p}_{{\rm{global}}}^{{\rm{MM}}}$) was retained.

The corrected global prior (${\widetilde{{\rm{p}}}}_{{\rm{global}}}$) was neither renormalized using softmax nor thresholded before being propagated to the FiLM conditioning module. Instead, it was used directly as a continuous conditioning vector. This design preserves the sigmoid-based multi-label formulation of the global model and avoids imposing a mutually exclusive probability simplex. Thresholding was applied only to the final task-specific sigmoid outputs during inference and evaluation.

Importantly, RAG correction modified only the first three primary-diagnostic dimensions of the 15-dimensional prior vector. The remaining dimensions corresponding to dementia etiologies and MCI subtypes were not directly overwritten by RAG at this stage. The downstream prior used for task-specific etiology and subtype inference was therefore assembled as:

3 $${\widetilde{p}}_{{\rm{prior}}}=[{\widetilde{p}}_{{\rm{global}}};{p}_{{\rm{down}}}^{{\rm{MM}}}]\in {[\mathrm{0,1}]}^{15}$$

where (${p}_{{\rm{down}}}^{{\rm{MM}}}\,\in \,{[\mathrm{0,1}]}^{12}$) denotes the original multimodal outputs for the downstream etiology and subtype dimensions. Thus, the role of RAG in this experiment was not to directly replace downstream predictions, but to adjust the upstream routing prior supplied to the hierarchical cascade under sparse-input conditions.

Final label assignment followed a predefined hierarchical logic. For each subject, the predicted global class was determined by the argmax of the corrected NC/MCI/DE scores. If the subject was assigned to NC, all etiology and subtype outputs were set to zero. If assigned to MCI, etiology outputs were suppressed and the MCI subtype label was assigned from the subtype branch. If assigned to DE, subtype outputs were suppressed and dementia etiology labels were binarized from the etiology branch probabilities. Performance of the original and RAG-corrected inference pipelines was then compared across all sparsity levels to evaluate whether knowledge-guided correction of the global routing prior could improve the robustness of hierarchical diagnosis under feature incompleteness.

### Physician review of report quality and clinical reasoning concordance

To assess the textual quality and clinical acceptability of MEDRF-generated reports, we conducted an independent physician review. A total of 80 cases were selected for evaluation, and each case was independently reviewed by five clinicians, yielding 400 case-level assessments in total. Summary statistics of the participating clinicians are provided in Supplementary Table 7. To ensure coverage of representative model–report behaviors, the reviewed cases were sampled to represent four descriptive scenarios defined by the relationship among the mHC prediction, the diagnosis stated in the generated report, and the reference diagnosis: (1) both the mHC prediction and the report diagnosis were correct (25 cases); (2) the mHC prediction was incorrect but the report diagnosis was concordant with the reference diagnosis (40 cases); (3) the mHC prediction was correct but the report diagnosis was incorrect (5 cases); (4) both were incorrect (10 cases).

Each clinician evaluated every report using a 5-point Likert-scale instrument covering five prespecified domains, with higher scores indicating better performance. The five domains were: factual accuracy, assessing whether the report faithfully reflected the available case information and avoided unsupported statements; diagnostic reasoning, assessing the coherence, plausibility, and logical consistency of the reasoning process leading to the stated conclusion; content completeness, assessing whether clinically relevant findings were sufficiently covered; clarity and professionalism, assessing readability, organization, wording, and appropriateness of clinical language; and clinical utility and safety, assessing whether the report would be useful in routine practice without introducing potentially misleading, unsafe, or overconfident recommendations.

In addition to domain-level scoring, clinicians recorded whether the diagnostic conclusion stated in the generated report was concordant with their own preliminary clinical judgment. Reviewers were also invited to provide free-text comments to highlight strengths, weaknesses, omissions, ambiguous wording, unsupported claims, and potential safety concerns. These qualitative comments were collated and summarized to identify recurrent themes, and representative verbatim feedback is provided in Supplementary Table 8.

Quantitative ratings were summarized using mean ± standard deviation, as well as median and interquartile range where appropriate. The internal consistency of the physician evaluation instrument was assessed using Cronbach’s α, with 95% confidence intervals calculated for the overall scale and for each individual domain.

## Supplementary information

Supplementary Information

## Data Availability

SCAN (MRI data collection from the NACC) and ADNI can be obtained from the LONI website at IDA - Image and Data Archive.NACC and OASIS-3 data can be downloaded at https://naccdata.org and https://sites.wustl.edu/oasisbrains/home/oasis-3/. The literature datasets and full study protocol used and analyzed during the current study are available from the corresponding author upon reasonable request.

## Code availability

The code used for model training, evaluation, and statistical analysis is publicly available on GitHub at https://github.com/hu1116/A-Multimodal-Evidence-Driven-Framework-for-Clinical-Decision-Support-in-Cognitive-Impairment.
