# Supplementary material for: A multimodal evidence-driven framework for clinical decision support in cognitive impairment
Source: NPJ Digit Med. 2026 Jul 20;9:879. doi: 10.1038/s41746-026-03048-9 (PMC13751622; doi:10.1038/s41746-026-03048-9)
Supplement: Supplementary file 1 [file 41746_2026_3048_MOESM1_ESM.pdf]

Supplementary Material

Supplementary Figures

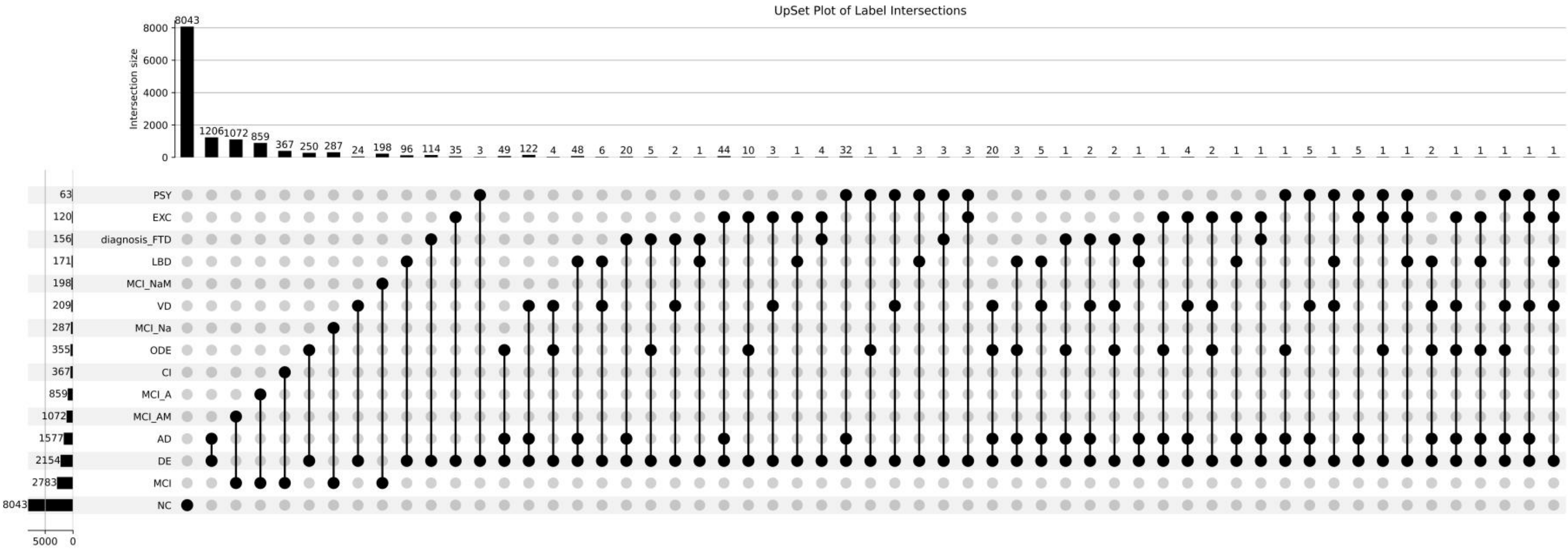

Supplementary figure 1. Distribution and hierarchical co-occurrence of diagnostic labels in the training cohort. The UpSet plot visualizes the frequency and intersection patterns of 15 diagnostic labels across the aggregated dataset

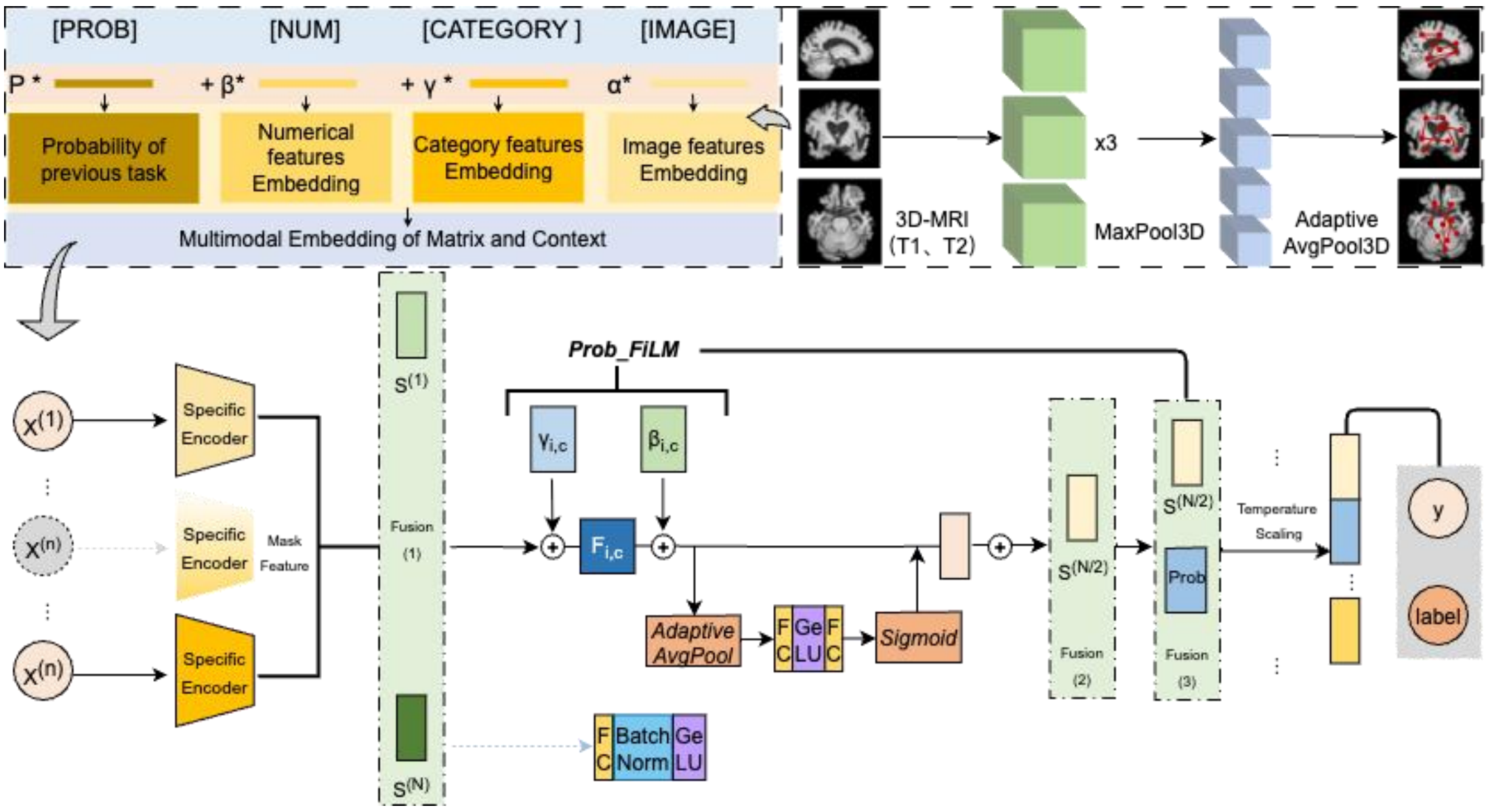

Supplementary figure 2. Probability-conditioned Multimodal Hierarchical Cascade classifier for cognitive staging. Posterior scores from the previous diagnostic tier are fused with tabular (numerical + categorical) and 3D-CNN embeddings; a FiLM block uses these scores to rescale the joint feature map, after which a temperature-scaled head outputs labels.

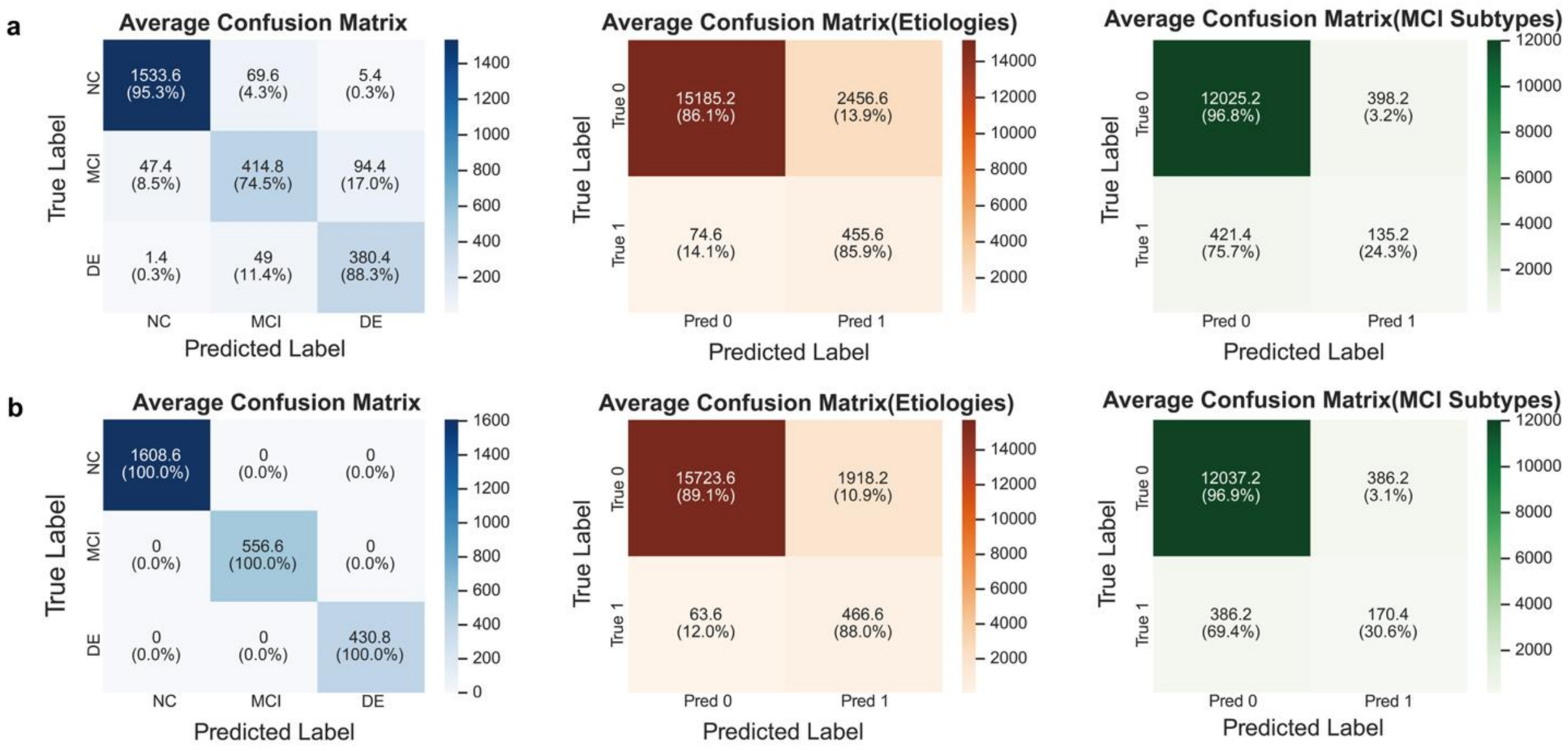

Supplementary figure 3. Analysis of cascading error across hierarchical transitions in the mHC. a, Confusion matrices represent averages across five cross-validation folds, with cell values indicating mean sample counts and row-normalized percentages reflecting per-class recall. For etiology and MCI subtype branches, all individual labels are pooled and evaluated as binary outcomes (Pred 0 / Pred 1), such that each patient may contribute multiple entries. a, Standard hierarchical routing using predicted primary state assignments. b, Oracle control in which downstream branches receive ground-truth upstream labels, isolating the contribution of routing errors to overall hierarchical error. The limited performance gains observed in b relative to a suggest that cascading errors contributed modestly to downstream mistakes, with the majority of errors reflecting intrinsic ambiguity in clinically overlapping cases.

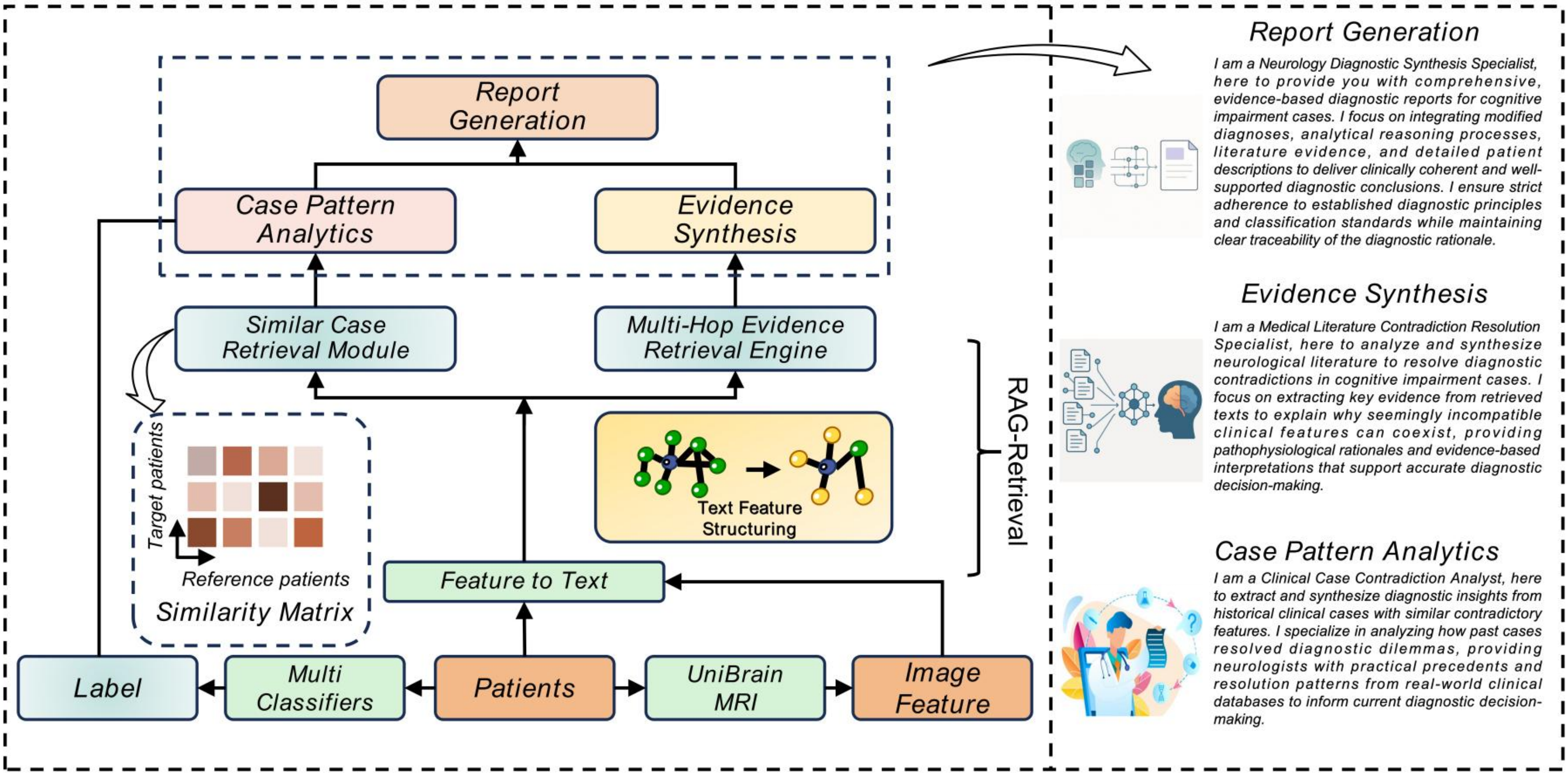

Supplementary figure 4. LLM-based retrieval-augmented generation for evidence-grounded diagnosis. An architecture synthesizes diagnostic evidence from multimodal clinical data. Feature-to-text transformation structures inputs for dual retrieval: population-level case matching and iterative literature mining. This collaboration resolves contradictory features through case pattern analysis and evidence synthesis, generating reports with traceable reasoning chains.

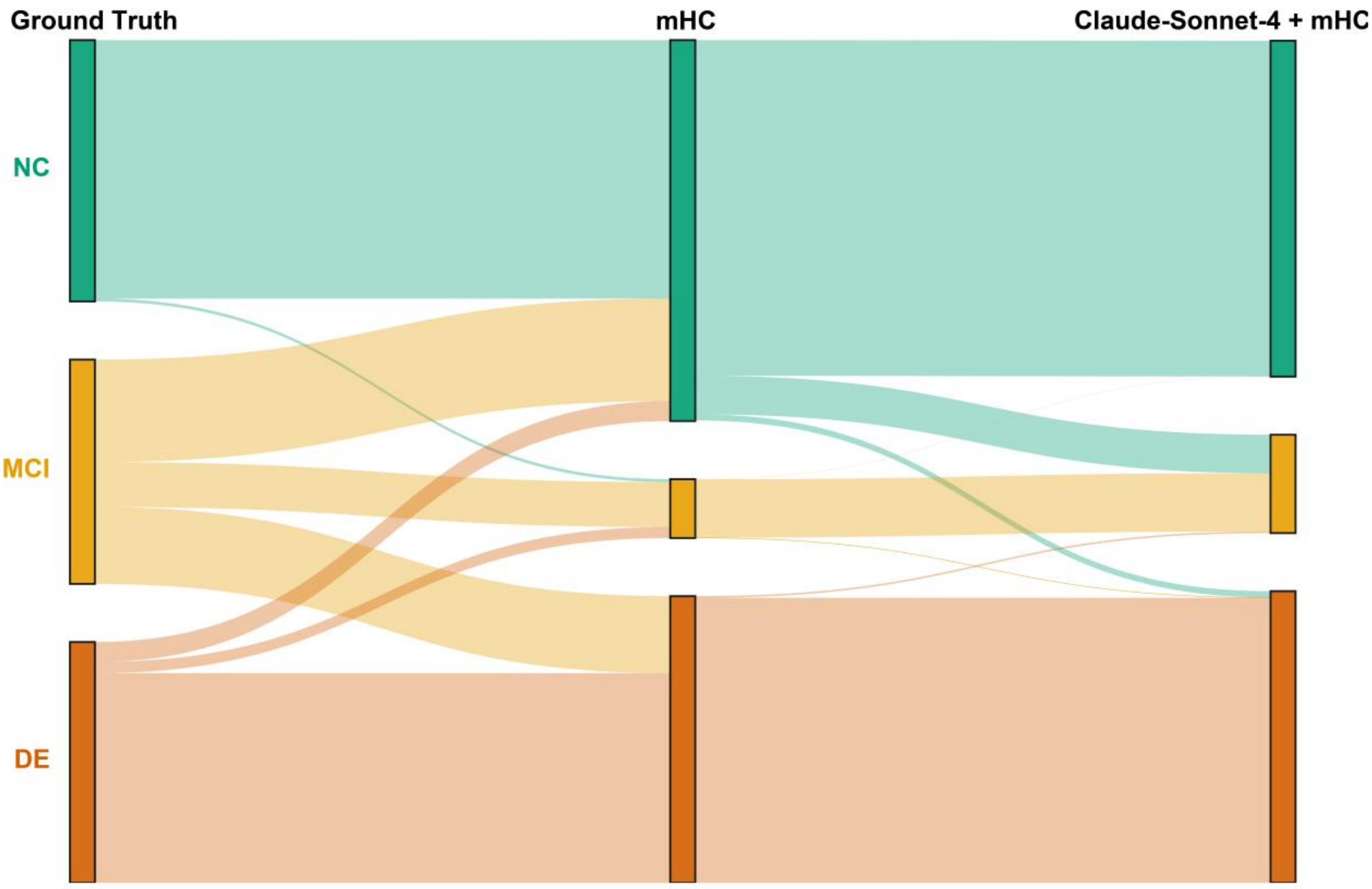

Supplementary figure 5. Diagnostic flow and consistency analysis across model stages. A Sankey diagram visualizing the transitions of patient classifications from Ground Truth (left) to the mHC model predictions (center), and finally to the Claude-Sonnet-4 integrated diagnosis (right). The width of the flows is proportional to the number of cases. Color coding represents the three primary states: Normal Control (NC, green), Mild Cognitive Impairment (MCI, yellow), and Dementia (DE, orange). The first transition (Left to Center) highlights the classification accuracy and error distribution of the mHC backbone (e.g., MCI cases misclassified as NC or DE). The second transition (Center to Right) demonstrates the high fidelity and stability of the LLM + mHC.

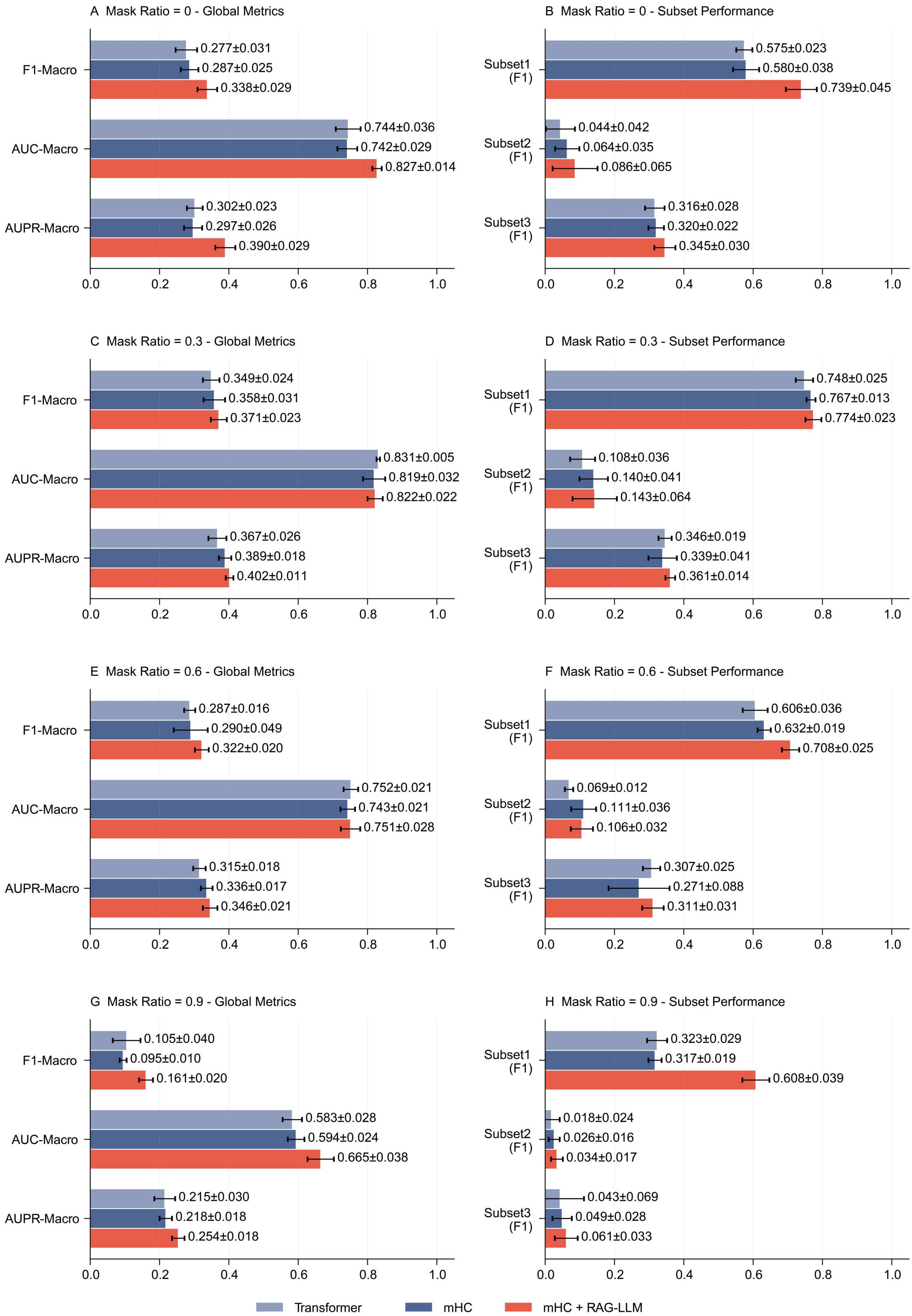

Supplementary figure 6. Performance of different models under progressively increased feature sparsity. Performance comparison of the Multimodal Transformer, mHC, and mHC + RAG-LLM under tabular feature masking ratios of 0, 0.3, 0.6, and 0.9. Panels A, C, and E report global metrics (macro-F1, macro-AUC, and macro-AUPR), and panels B, D, and F report subset-level F1 scores for Subset 1 (Primary State), Subset 2 (MCI Subtype), and Subset 3 (Etiology). Error bars denote standard deviation across folds. Performance decreased with increasing sparsity in all models. The benefit of RAG-LLM correction became

increasingly evident at higher masking levels, with the largest gains observed in Subset 1, while improvements in downstream subsets were smaller and more variable.

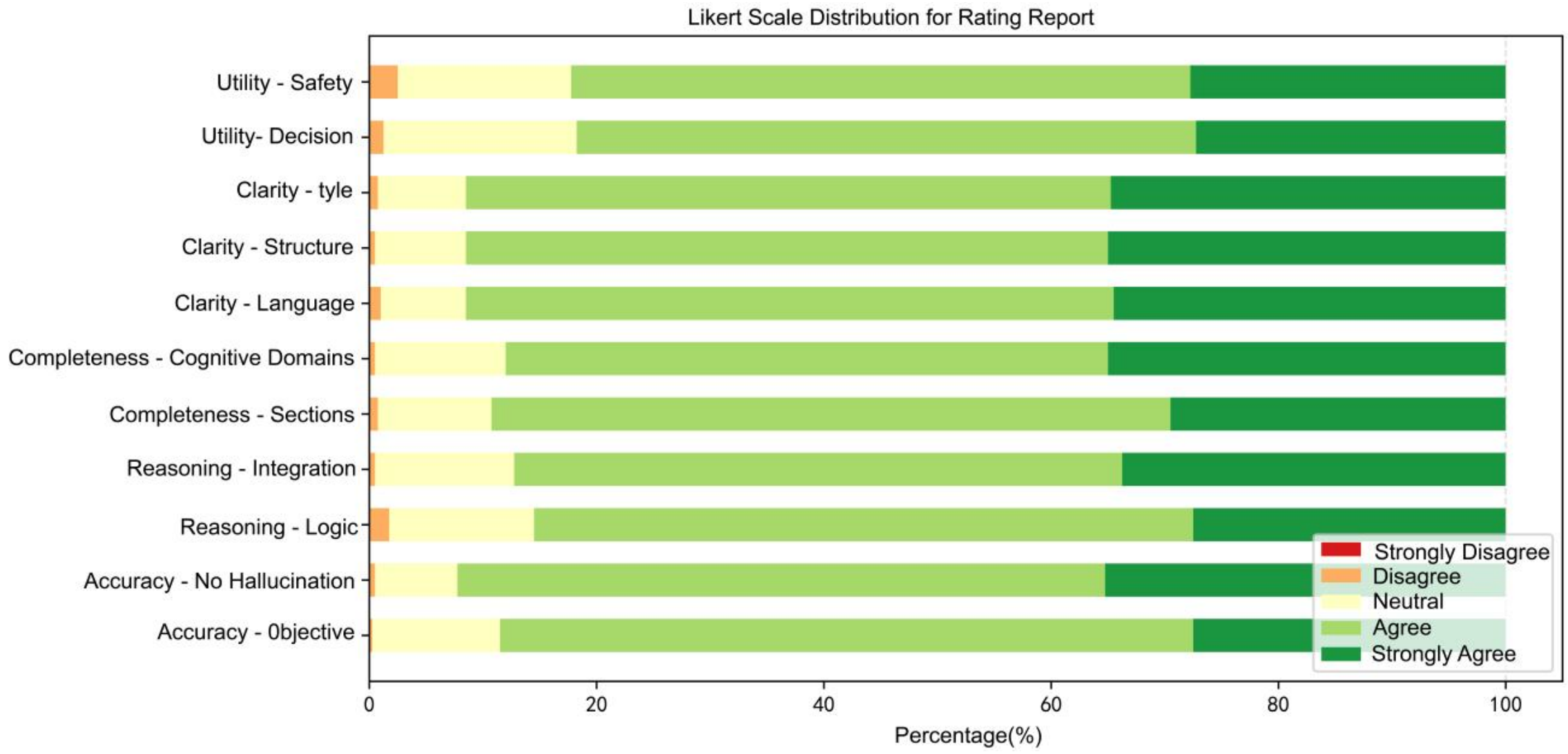

Supplementary figure 7. Likert scale distribution of evaluation ratings across key performance dimensions. The stacked bar chart illustrates the percentage distribution of expert/user ratings for the generated reports based on a 5-point Likert scale, ranging from "Strongly Disagree" (red) to "Strongly Agree" (dark green). The evaluation encompasses five primary domains, which are further categorized into specific sub-dimensions: Utility (Safety, Decision), Clarity (Style*, Structure, Language), Completeness (Cognitive Domains, Sections), Reasoning (Integration, Logic), and Accuracy (No Hallucination, Objective). The predominant proportion of "Agree" and "Strongly Agree" responses across all criteria indicates a highly positive consensus regarding the overall quality and reliability of the reports. Negative responses ("Disagree" and "Strongly Disagree") are negligible across all evaluated metrics.

Supplementary Tables

Supplementary Table 1. Performance of single-modal and multimodal classifiers on the NACC&OASIS training cohorts using five-fold cross-validation.

Comparison model	Accuracy	Precision	Recall	Specificity	F1 Score	MCC	AUROC	AUPR
Single modality Multi-label Classification (Image)								
NC	0.585 ± 0.028	0.686 ± 0.052	0.672 ± 0.245	0.443 ± 0.328	**0.647 ± 0.093**	0.120 ± 0.078	0.597 ± 0.032	0.695 ± 0.019
MCI	0.454 ± 0.109	0.242 ± 0.014	0.721 ± 0.198	0.381 ± 0.189	**0.356 ± 0.038**	0.092 ± 0.050	0.580 ± 0.037	0.260 ± 0.026
DE	0.533 ± 0.045	0.202 ± 0.007	0.618 ± 0.107	0.516 ± 0.074	**0.303 ± 0.020**	0.101 ± 0.029	0.581 ± 0.022	0.209 ± 0.018
CI	0.508 ± 0.262	0.033 ± 0.002	0.563 ± 0.286	0.506 ± 0.278	**0.060 ± 0.005**	0.028 ± 0.008	0.535 ± 0.012	0.033 ± 0.002
MCI_A	0.433 ± 0.089	0.080 ± 0.005	0.716 ± 0.126	0.413 ± 0.104	**0.143 ± 0.008**	0.067 ± 0.027	0.585 ± 0.023	0.086 ± 0.011
MCI_AM	0.435 ± 0.119	0.093 ± 0.009	0.663 ± 0.162	0.414 ± 0.143	**0.162 ± 0.014**	0.046 ± 0.038	0.565 ± 0.054	0.105 ± 0.017
MCI_Na	0.353 ± 0.091	0.028 ± 0.001	0.837 ± 0.099	0.342 ± 0.095	**0.054 ± 0.003**	0.057 ± 0.009	0.621 ± 0.013	0.033 ± 0.004
MCI_NaM	0.366 ± 0.166	0.019 ± 0.002	0.805 ± 0.172	0.360 ± 0.171	**0.038 ± 0.003**	0.045 ± 0.011	0.609 ± 0.052	0.023 ± 0.004
AD	0.549 ± 0.073	0.170 ± 0.008	0.692 ± 0.130	0.529 ± 0.100	**0.271 ± 0.014**	0.147 ± 0.027	0.625 ± 0.028	0.173 ± 0.013
LBD	0.501 ± 0.084	0.021 ± 0.001	0.818 ± 0.118	0.496 ± 0.086	**0.042 ± 0.003**	0.073 ± 0.011	0.685 ± 0.017	0.024 ± 0.004
VD	0.335 ± 0.124	0.020 ± 0.003	0.816 ± 0.109	0.327 ± 0.127	**0.039 ± 0.005**	0.039 ± 0.028	0.616 ± 0.072	0.030 ± 0.007
FTD	0.404 ± 0.145	0.018 ± 0.002	0.860 ± 0.109	0.399 ± 0.148	**0.035 ± 0.005**	0.060 ± 0.010	0.667 ± 0.044	0.033 ± 0.014
EXC	0.498 ± 0.195	0.011 ± 0.004	0.551 ± 0.147	0.497 ± 0.198	**0.022 ± 0.007**	0.011 ± 0.028	0.544 ± 0.070	0.013 ± 0.003
PSY	0.529 ± 0.305	0.009 ± 0.004	0.666 ± 0.249	0.529 ± 0.308	**0.017 ± 0.008**	0.017 ± 0.054	0.652 ± 0.089	0.013 ± 0.006
ODE	0.581 ± 0.138	0.051 ± 0.018	0.721 ± 0.049	0.577 ± 0.142	**0.095 ± 0.030**	0.105 ± 0.049	0.694 ± 0.050	0.072 ± 0.021
Single modality Multi-label Classification (Tabular)								
NC	0.943 ± 0.008	0.976 ± 0.003	0.930 ± 0.012	0.963 ± 0.004	**0.953 ± 0.007**	0.882 ± 0.015	0.984 ± 0.002	0.989 ± 0.002
MCI	0.854 ± 0.021	0.609 ± 0.040	0.911 ± 0.013	0.839 ± 0.027	**0.729 ± 0.028**	0.660 ± 0.034	0.936 ± 0.010	0.754 ± 0.049
DE	0.915 ± 0.008	0.669 ± 0.023	0.971 ± 0.012	0.904 ± 0.010	**0.792 ± 0.016**	0.761 ± 0.018	0.982 ± 0.003	0.914 ± 0.012
CI	0.707 ± 0.015	0.073 ± 0.005	0.793 ± 0.034	0.705 ± 0.016	**0.133 ± 0.009**	0.179 ± 0.016	0.809 ± 0.015	0.136 ± 0.018
MCI_A	0.743 ± 0.021	0.200 ± 0.012	0.955 ± 0.016	0.728 ± 0.023	**0.330 ± 0.017**	0.365 ± 0.017	0.889 ± 0.009	0.260 ± 0.022
MCI_AM	0.741 ± 0.023	0.237 ± 0.017	0.957 ± 0.005	0.721 ± 0.025	**0.380 ± 0.022**	0.397 ± 0.023	0.889 ± 0.013	0.329 ± 0.036
MCI_Na	0.695 ± 0.030	0.066 ± 0.007	0.955 ± 0.021	0.689 ± 0.031	**0.122 ± 0.011**	0.203 ± 0.016	0.882 ± 0.009	0.113 ± 0.013
MCI_NaM	0.679 ± 0.030	0.045 ± 0.004	0.985 ± 0.012	0.674 ± 0.030	**0.086 ± 0.008**	0.172 ± 0.012	0.892 ± 0.020	0.101 ± 0.030
AD	0.873 ± 0.015	0.490 ± 0.029	0.983 ± 0.009	0.857 ± 0.017	**0.653 ± 0.026**	0.640 ± 0.025	0.969 ± 0.005	0.738 ± 0.019
LBD	0.806 ± 0.018	0.064 ± 0.006	0.988 ± 0.015	0.804 ± 0.018	**0.119 ± 0.010**	0.224 ± 0.013	0.965 ± 0.005	0.304 ± 0.071
VD	0.754 ± 0.024	0.061 ± 0.006	0.976 ± 0.021	0.751 ± 0.025	**0.114 ± 0.011**	0.209 ± 0.014	0.910 ± 0.012	0.098 ± 0.014
FTD	0.750 ± 0.019	0.044 ± 0.004	0.955 ± 0.026	0.747 ± 0.020	**0.084 ± 0.008**	0.175 ± 0.013	0.907 ± 0.005	0.112 ± 0.021
EXC	0.752 ± 0.013	0.036 ± 0.004	0.983 ± 0.022	0.750 ± 0.013	**0.069 ± 0.007**	0.161 ± 0.011	0.905 ± 0.011	0.061 ± 0.010
PSY	0.723 ± 0.038	0.016 ± 0.002	0.920 ± 0.053	0.722 ± 0.039	**0.032 ± 0.004**	0.100 ± 0.011	0.896 ± 0.014	0.042 ± 0.013
ODE	0.814 ± 0.022	0.125 ± 0.012	0.961 ± 0.020	0.810 ± 0.022	**0.222 ± 0.020**	0.309 ± 0.021	0.953 ± 0.007	0.511 ± 0.078
Multimodality Multi-label Classification (Image + Tabular, Catboost)								
NC	0.954 ± 0.004	0.965 ± 0.001	0.961 ± 0.007	0.943 ± 0.002	**0.963 ± 0.004**	0.903 ± 0.009	0.984 ± 0.002	0.988 ± 0.002
MCI	0.856 ± 0.008	0.617 ± 0.018	0.874 ± 0.023	0.851 ± 0.014	**0.723 ± 0.008**	0.647 ± 0.010	0.934 ± 0.004	0.782 ± 0.021
DE	0.899 ± 0.004	0.631 ± 0.010	0.947 ± 0.007	0.890 ± 0.004	**0.757 ± 0.008**	0.720 ± 0.010	0.977 ± 0.001	0.892 ± 0.009
CI	0.735 ± 0.035	0.073 ± 0.006	0.716 ± 0.100	0.736 ± 0.039	**0.133 ± 0.010**	0.168 ± 0.022	0.803 ± 0.025	0.140 ± 0.043
MCI_A	0.734 ± 0.006	0.192 ± 0.005	0.935 ± 0.012	0.720 ± 0.006	**0.318 ± 0.008**	0.348 ± 0.010	0.916 ± 0.004	0.388 ± 0.030
MCI_AM	0.739 ± 0.007	0.229 ± 0.005	0.912 ± 0.015	0.723 ± 0.007	**0.366 ± 0.007**	0.372 ± 0.010	0.899 ± 0.006	0.381 ± 0.039
MCI_Na	0.688 ± 0.007	0.060 ± 0.003	0.889 ± 0.039	0.684 ± 0.006	**0.112 ± 0.006**	0.179 ± 0.014	0.896 ± 0.019	0.205 ± 0.038
MCI_NaM	0.734 ± 0.008	0.045 ± 0.004	0.813 ± 0.074	0.733 ± 0.008	**0.085 ± 0.007**	0.150 ± 0.019	0.878 ± 0.022	0.134 ± 0.025
AD	0.874 ± 0.005	0.492 ± 0.010	0.962 ± 0.004	0.862 ± 0.006	**0.651 ± 0.009**	0.633 ± 0.009	0.968 ± 0.002	0.779 ± 0.025
LBD	0.921 ± 0.007	0.138 ± 0.017	0.936 ± 0.051	0.921 ± 0.007	**0.240 ± 0.027**	0.342 ± 0.033	0.973 ± 0.013	0.475 ± 0.097
VD	0.773 ± 0.003	0.062 ± 0.002	0.919 ± 0.032	0.771 ± 0.003	**0.115 ± 0.004**	0.203 ± 0.008	0.898 ± 0.017	0.148 ± 0.051
FTD	0.828 ± 0.009	0.062 ± 0.004	0.943 ± 0.060	0.826 ± 0.010	**0.116 ± 0.006**	0.217 ± 0.013	0.957 ± 0.012	0.312 ± 0.116
EXC	0.760 ± 0.008	0.035 ± 0.003	0.934 ± 0.019	0.758 ± 0.007	**0.067 ± 0.006**	0.153 ± 0.009	0.906 ± 0.014	0.101 ± 0.038
PSY	0.829 ± 0.027	0.025 ± 0.002	0.887 ± 0.114	0.829 ± 0.028	**0.048 ± 0.004**	0.131 ± 0.013	0.933 ± 0.025	0.128 ± 0.049
ODE	0.846 ± 0.015	0.142 ± 0.014	0.913 ± 0.021	0.844 ± 0.015	**0.246 ± 0.022**	0.324 ± 0.023	0.954 ± 0.007	0.603 ± 0.045

Multimodality Multi-label Classification (Image + Tabular, Joint Learning Strategy，JLS)								
NC	0.953 ± 0.005	0.969 ± 0.002	0.955 ± 0.010	0.950 ± 0.004	**0.962 ± 0.004**	0.901 ± 0.010	0.968 ± 0.004	0.981 ± 0.002
MCI	0.900 ± 0.007	0.752 ± 0.023	0.794 ± 0.019	0.928 ± 0.009	**0.773 ± 0.015**	0.709 ± 0.019	0.939 ± 0.006	0.779 ± 0.029
DE	0.940 ± 0.004	0.769 ± 0.015	0.910 ± 0.013	0.946 ± 0.005	**0.833 ± 0.009**	0.801 ± 0.010	0.979 ± 0.002	0.899 ± 0.010
CI	0.780 ± 0.011	0.077 ± 0.004	0.613 ± 0.045	0.785 ± 0.012	**0.136 ± 0.008**	0.158 ± 0.015	0.764 ± 0.020	0.085 ± 0.010
MCI_A	0.820 ± 0.010	0.248 ± 0.012	0.844 ± 0.031	0.818 ± 0.010	**0.383 ± 0.016**	0.394 ± 0.020	0.877 ± 0.011	0.223 ± 0.011
MCI_AM	0.824 ± 0.007	0.297 ± 0.007	0.823 ± 0.021	0.824 ± 0.009	**0.436 ± 0.005**	0.424 ± 0.005	0.886 ± 0.006	0.307 ± 0.021
MCI_Na	0.795 ± 0.008	0.086 ± 0.003	0.857 ± 0.040	0.794 ± 0.009	**0.156 ± 0.004**	0.231 ± 0.010	0.900 ± 0.008	0.152 ± 0.032
MCI_NaM	0.789 ± 0.009	0.057 ± 0.001	0.833 ± 0.052	0.788 ± 0.010	**0.107 ± 0.002**	0.183 ± 0.009	0.888 ± 0.016	0.115 ± 0.012
AD	0.904 ± 0.005	0.566 ± 0.015	0.912 ± 0.011	0.903 ± 0.006	**0.698 ± 0.013**	0.671 ± 0.014	0.959 ± 0.004	0.678 ± 0.029
LBD	0.828 ± 0.011	0.069 ± 0.005	0.954 ± 0.030	0.827 ± 0.011	**0.128 ± 0.009**	0.230 ± 0.011	0.949 ± 0.010	0.176 ± 0.058
VD	0.820 ± 0.006	0.073 ± 0.003	0.872 ± 0.074	0.819 ± 0.007	**0.135 ± 0.006**	0.220 ± 0.018	0.899 ± 0.011	0.082 ± 0.010
FTD	0.819 ± 0.011	0.053 ± 0.004	0.834 ± 0.062	0.819 ± 0.012	**0.100 ± 0.008**	0.182 ± 0.016	0.910 ± 0.007	0.098 ± 0.010
EXC	0.816 ± 0.007	0.043 ± 0.006	0.882 ± 0.067	0.815 ± 0.007	**0.081 ± 0.010**	0.170 ± 0.021	0.893 ± 0.019	0.053 ± 0.011
PSY	0.817 ± 0.010	0.022 ± 0.002	0.857 ± 0.061	0.817 ± 0.010	**0.044 ± 0.003**	0.120 ± 0.011	0.889 ± 0.013	0.029 ± 0.005
ODE	0.834 ± 0.007	0.136 ± 0.006	0.944 ± 0.013	0.831 ± 0.007	**0.238 ± 0.009**	0.322 ± 0.009	0.901 ± 0.011	0.135 ± 0.020
Multimodality Multi-label Classification (Image + Tabular, transformer)								
NC	0.949 ± 0.005	0.967 ± 0.001	0.950 ± 0.008	0.947 ± 0.002	**0.958 ± 0.004**	0.892 ± 0.009	0.971 ± 0.004	0.979 ± 0.003
MCI	0.885 ± 0.006	0.707 ± 0.019	0.797 ± 0.017	0.909 ± 0.009	**0.749 ± 0.011**	0.677 ± 0.014	0.917 ± 0.006	0.705 ± 0.014
DE	0.929 ± 0.004	0.737 ± 0.012	0.890 ± 0.021	0.937 ± 0.004	**0.806 ± 0.011**	0.768 ± 0.014	0.971 ± 0.005	0.842 ± 0.024
CI	0.795 ± 0.010	0.081 ± 0.003	0.605 ± 0.031	0.801 ± 0.011	**0.143 ± 0.005**	0.165 ± 0.008	0.727 ± 0.014	0.110 ± 0.029
MCI_A	0.813 ± 0.006	0.239 ± 0.005	0.838 ± 0.019	0.811 ± 0.007	**0.372 ± 0.006**	0.382 ± 0.008	0.882 ± 0.006	0.244 ± 0.018
MCI_AM	0.809 ± 0.008	0.279 ± 0.009	0.830 ± 0.023	0.807 ± 0.009	**0.418 ± 0.010**	0.407 ± 0.013	0.875 ± 0.009	0.310 ± 0.024
MCI_Na	0.792 ± 0.012	0.085 ± 0.003	0.854 ± 0.041	0.791 ± 0.013	**0.154 ± 0.005**	0.228 ± 0.009	0.878 ± 0.009	0.127 ± 0.035
MCI_NaM	0.782 ± 0.010	0.056 ± 0.004	0.828 ± 0.025	0.782 ± 0.010	**0.104 ± 0.008**	0.178 ± 0.013	0.853 ± 0.028	0.087 ± 0.028
AD	0.914 ± 0.002	0.600 ± 0.009	0.868 ± 0.023	0.920 ± 0.004	**0.710 ± 0.009**	0.677 ± 0.011	0.952 ± 0.008	0.630 ± 0.037
LBD	0.864 ± 0.018	0.083 ± 0.010	0.907 ± 0.051	0.864 ± 0.019	**0.151 ± 0.017**	0.250 ± 0.019	0.950 ± 0.017	0.322 ± 0.156
VD	0.829 ± 0.013	0.072 ± 0.005	0.800 ± 0.047	0.830 ± 0.013	**0.132 ± 0.009**	0.206 ± 0.014	0.884 ± 0.009	0.085 ± 0.017
FTD	0.844 ± 0.005	0.060 ± 0.002	0.821 ± 0.029	0.844 ± 0.005	**0.112 ± 0.003**	0.196 ± 0.008	0.912 ± 0.011	0.092 ± 0.018
EXC	0.818 ± 0.010	0.039 ± 0.005	0.797 ± 0.111	0.818 ± 0.011	**0.074 ± 0.009**	0.150 ± 0.022	0.883 ± 0.031	0.059 ± 0.017
PSY	0.838 ± 0.006	0.024 ± 0.003	0.810 ± 0.064	0.839 ± 0.006	**0.046 ± 0.005**	0.122 ± 0.012	0.908 ± 0.021	0.069 ± 0.050
ODE	0.870 ± 0.008	0.159 ± 0.006	0.873 ± 0.032	0.870 ± 0.009	**0.270 ± 0.009**	0.340 ± 0.011	0.940 ± 0.013	0.304 ± 0.105

Supplementary Table 2. Cascaded multimodal fusion network classifier performance from five-fold cross-validation. This table presents the performance metrics of our mHC model across the NACC + OASIS datasets.

Classifier	Accuracy	Precision	Recall	Specificity	F1 Score	MCC	AUROC	AUPR
Multimodality Two-tier Labeling System (Image + Tabular, mHC)								
NC	0.952 ± 0.006	0.969 ± 0.002	0.953 ± 0.011	0.951 ± 0.004	0.961 ± 0.005	0.900 ± 0.011	0.968 ± 0.004	0.981 ± 0.002
MCI	0.900 ± 0.006	0.778 ± 0.024	0.745 ± 0.011	0.942 ± 0.008	0.761 ± 0.011	0.698 ± 0.016	0.939 ± 0.006	0.779 ± 0.029
DE	0.942 ± 0.003	0.792 ± 0.011	0.883 ± 0.011	0.954 ± 0.003	0.835 ± 0.007	0.802 ± 0.008	0.979 ± 0.002	0.897 ± 0.012
CI	0.969 ± 0.004	0.080 ± 0.111	0.046 ± 0.079	0.996 ± 0.006	0.056 ± 0.092	0.053 ± 0.086	0.717 ± 0.067	0.097 ± 0.032
MCI_A	0.915 ± 0.015	0.295 ± 0.150	0.367 ± 0.204	0.954 ± 0.030	0.320 ± 0.161	0.288 ± 0.145	0.878 ± 0.040	0.338 ± 0.053
MCI_AM	0.888 ± 0.015	0.363 ± 0.033	0.418 ± 0.133	0.931 ± 0.028	0.372 ± 0.050	0.323 ± 0.045	0.887 ± 0.010	0.358 ± 0.037
MCI_Na	0.974 ± 0.007	0.044 ± 0.088	0.067 ± 0.133	0.995 ± 0.010	0.053 ± 0.106	0.049 ± 0.101	0.835 ± 0.036	0.117 ± 0.054
MCI_NaM	0.962 ± 0.019	0.075 ± 0.045	0.147 ± 0.085	0.974 ± 0.021	0.094 ± 0.051	0.086 ± 0.046	0.848 ± 0.026	0.082 ± 0.012
AD	0.933 ± 0.005	0.678 ± 0.024	0.856 ± 0.011	0.944 ± 0.006	**0.757 ± 0.015**	0.725 ± 0.016	0.967 ± 0.002	0.813 ± 0.010
LBD	0.936 ± 0.016	0.164 ± 0.034	0.883 ± 0.018	0.937 ± 0.016	**0.275 ± 0.048**	0.363 ± 0.042	0.979 ± 0.003	0.576 ± 0.079
VD	0.866 ± 0.056	0.090 ± 0.034	0.658 ± 0.276	0.870 ± 0.062	**0.140 ± 0.016**	0.195 ± 0.034	0.874 ± 0.060	0.193 ± 0.103
FTD	0.910 ± 0.047	0.078 ± 0.016	0.527 ± 0.276	0.915 ± 0.051	**0.119 ± 0.027**	0.165 ± 0.052	0.877 ± 0.058	0.123 ± 0.059
EXC	0.856 ± 0.062	0.047 ± 0.009	0.704 ± 0.319	0.857 ± 0.066	**0.078 ± 0.007**	0.146 ± 0.045	0.796 ± 0.133	0.059 ± 0.020
PSY	0.896 ± 0.047	0.035 ± 0.009	0.684 ± 0.231	0.897 ± 0.048	**0.065 ± 0.012**	0.134 ± 0.021	0.880 ± 0.064	0.080 ± 0.047
ODE	0.897 ± 0.032	0.206 ± 0.082	0.781 ± 0.099	0.900 ± 0.036	**0.311 ± 0.072**	0.359 ± 0.046	0.917 ± 0.024	0.426 ± 0.049

Supplementary Table 3. LLM + Classifier performance from five-fold cross-validation. This table presents the performance metrics of our structure across the external validation (ADNI).

Dataset (ADNI)	Precision	Recall	Specificity	F1 Score	MCC
NC					
Claude-Sonnet-4 + mHC	0.771 ± 0.060	0.985 ± 0.009	0.831 ± 0.062	**0.864 ± 0.036**	0.788 ± 0.055
DeepSeek-R1 + mHC	0.855 ± 0.013	0.851 ± 0.005	0.919 ± 0.009	**0.853 ± 0.005**	0.771 ± 0.010
GPT-5 + mHC	0.702 ± 0.063	0.989 ± 0.009	0.758 ± 0.078	**0.819 ± 0.041**	0.720 ± 0.063
mHC	0.684 ± 0.068	0.989 ± 0.008	0.819 ± 0.043	**0.721 ± 0.066**	0.758 ± 0.087
Claude-Sonnet-4	0.851 ± 0.006	0.675 ± 0.017	0.934 ± 0.002	**0.753 ± 0.013**	0.647 ± 0.016
MCI					
Claude-Sonnet-4 + mHC	0.775 ± 0.020	0.341 ± 0.092	0.957 ± 0.008	**0.469 ± 0.097**	0.399 ± 0.074
DeepSeek-R1 + mHC	0.603 ± 0.011	0.523 ± 0.036	0.846 ± 0.005	**0.560 ± 0.025**	0.385 ± 0.027
GPT-5 + mHC	0.761 ± 0.013	0.230 ± 0.098	0.968 ± 0.013	**0.344 ± 0.129**	0.308 ± 0.086
mHC	0.768 ± 0.010	0.200 ± 0.084	0.972 ± 0.013	**0.285 ± 0.125**	0.282 ± 0.093
Claude-Sonnet-4	0.473 ± 0.009	0.695 ± 0.024	0.654 ± 0.006	**0.563 ± 0.014**	0.324 ± 0.022
DE					
Claude-Sonnet-4 + mHC	0.731 ± 0.026	0.884 ± 0.031	0.838 ± 0.026	**0.799 ± 0.014**	0.694 ± 0.021
DeepSeek-R1 + mHC	0.740 ± 0.018	0.824 ± 0.012	0.856 ± 0.014	**0.779 ± 0.010**	0.663 ± 0.016
GPT-5 + mHC	0.737 ± 0.028	0.879 ± 0.031	0.844 ± 0.026	**0.802 ± 0.013**	0.697 ± 0.020
mHC	0.733 ± 0.022	0.871 ± 0.038	0.842 ± 0.023	**0.795 ± 0.014**	0.687 ± 0.023
Claude-Sonnet-4	0.785 ± 0.023	0.595 ± 0.014	0.919 ± 0.012	**0.677 ± 0.009**	0.558 ± 0.015

Supplementary Table 4. Label-specific classification performance for primary diagnostic categories (NC, MCI, DE) under progressive feature sparsity with RAG_LLM guided.

Comparison model	Accuracy	Precision	Recall	Specificity	F1 Score	MCC	AUROC	AUPR
**NC (mask ratio = 0)**								
Transformer	0.819 ± 0.014	0.752 ± 0.015	0.998 ± 0.005	0.607 ± 0.032	**0.857 ± 0.010**	0.672 ± 0.025	0.917 ± 0.046	0.877 ± 0.063
mHC	0.821 ± 0.017	0.753 ± 0.017	0.998 ± 0.005	0.610 ± 0.037	**0.858 ± 0.011**	0.674 ± 0.029	0.902 ± 0.043	0.871 ± 0.063
mHC + RAG-LLM	0.902 ± 0.026	0.894 ± 0.036	0.933 ± 0.022	0.866 ± 0.049	**0.912 ± 0.022**	0.804 ± 0.052	0.964 ± 0.015	0.958 ± 0.021
**MCI (mask ratio = 0)**								
Transformer	0.815 ± 0.008	0.283 ± 0.067	0.073 ± 0.040	0.964 ± 0.013	**0.113 ± 0.054**	0.066 ± 0.054	0.618 ± 0.059	0.253 ± 0.037
mHC	0.807 ± 0.020	0.260 ± 0.141	0.082 ± 0.059	0.953 ± 0.022	**0.120 ± 0.080**	0.056 ± 0.090	0.614 ± 0.046	0.247 ± 0.028
mHC + RAG-LLM	0.817 ± 0.029	0.460 ± 0.069	0.571 ± 0.101	0.866 ± 0.017	**0.509 ± 0.080**	0.402 ± 0.100	0.820 ± 0.052	0.450 ± 0.111
**DE (mask ratio = 0)**								
Transformer	0.871 ± 0.019	0.839 ± 0.031	0.686 ± 0.057	0.947 ± 0.012	**0.753 ± 0.042**	0.675 ± 0.049	0.914 ± 0.038	0.839 ± 0.057
mHC	0.875 ± 0.021	0.846 ± 0.035	0.695 ± 0.063	0.948 ± 0.013	**0.761 ± 0.048**	0.685 ± 0.056	0.916 ± 0.033	0.831 ± 0.052
mHC + RAG-LLM	0.895 ± 0.019	0.912 ± 0.050	0.709 ± 0.045	0.971 ± 0.017	**0.796 ± 0.038**	0.739 ± 0.050	0.968 ± 0.014	0.932 ± 0.019
**NC (mask ratio = 0.3)**								
Transformer	0.920 ± 0.013	0.913 ± 0.009	0.943 ± 0.019	0.893 ± 0.011	**0.927 ± 0.012**	0.839 ± 0.027	0.951 ± 0.009	0.948 ± 0.015
mHC	0.931 ± 0.010	0.933 ± 0.012	0.940 ± 0.017	0.920 ± 0.015	**0.937 ± 0.009**	0.861 ± 0.020	0.948 ± 0.012	0.952 ± 0.017
mHC + RAG-LLM	0.920 ± 0.019	0.948 ± 0.019	0.902 ± 0.029	0.941 ± 0.022	**0.924 ± 0.018**	0.841 ± 0.037	0.951 ± 0.013	0.956 ± 0.019
**MCI (mask ratio = 0.3)**								
Transformer	0.836 ± 0.024	0.513 ± 0.059	0.610 ± 0.032	0.881 ± 0.027	**0.556 ± 0.038**	0.460 ± 0.051	0.798 ± 0.020	0.450 ± 0.065
mHC	0.837 ± 0.013	0.512 ± 0.024	0.649 ± 0.067	0.874 ± 0.025	**0.570 ± 0.022**	0.479 ± 0.025	0.848 ± 0.032	0.569 ± 0.083
mHC + RAG-LLM	0.833 ± 0.018	0.502 ± 0.033	0.714 ± 0.072	0.856 ± 0.026	**0.588 ± 0.034**	0.501 ± 0.044	0.861 ± 0.019	0.585 ± 0.076
**DE (mask ratio = 0.3)**								
Transformer	0.889 ± 0.026	0.873 ± 0.036	0.718 ± 0.076	0.958 ± 0.010	**0.787 ± 0.059**	0.720 ± 0.071	0.949 ± 0.010	0.888 ± 0.022
mHC	0.893 ± 0.014	0.881 ± 0.028	0.727 ± 0.058	0.960 ± 0.011	**0.795 ± 0.036**	0.731 ± 0.041	0.964 ± 0.007	0.911 ± 0.021
mHC + RAG-LLM	0.899 ± 0.023	0.888 ± 0.042	0.746 ± 0.068	0.962 ± 0.014	**0.809 ± 0.050**	0.748 ± 0.062	0.964 ± 0.013	0.920 ± 0.026
**NC (mask ratio = 0.6)**								
Transformer	0.842 ± 0.021	0.787 ± 0.014	0.973 ± 0.026	0.687 ± 0.023	**0.870 ± 0.017**	0.700 ± 0.046	0.915 ± 0.025	0.903 ± 0.026
mHC	0.859 ± 0.028	0.820 ± 0.038	0.953 ± 0.009	0.748 ± 0.063	**0.880 ± 0.021**	0.724 ± 0.051	0.932 ± 0.028	0.923 ± 0.044
mHC + RAG-LLM	0.893 ± 0.020	0.911 ± 0.020	0.890 ± 0.029	0.896 ± 0.025	**0.900 ± 0.019**	0.785 ± 0.040	0.946 ± 0.025	0.938 ± 0.037
**MCI (mask ratio = 0.6)**								
Transformer	0.777 ± 0.032	0.359 ± 0.053	0.382 ± 0.044	0.857 ± 0.043	**0.366 ± 0.027**	0.235 ± 0.044	0.681 ± 0.031	0.314 ± 0.040
mHC	0.766 ± 0.011	0.350 ± 0.017	0.472 ± 0.087	0.825 ± 0.024	**0.400 ± 0.042**	0.265 ± 0.048	0.735 ± 0.053	0.384 ± 0.056
mHC + RAG-LLM	0.780 ± 0.017	0.400 ± 0.035	0.659 ± 0.126	0.804 ± 0.019	**0.497 ± 0.061**	0.387 ± 0.083	0.775 ± 0.074	0.428 ± 0.065
**DE (mask ratio = 0.6)**								
Transformer	0.821 ± 0.028	0.884 ± 0.066	0.442 ± 0.101	0.975 ± 0.013	**0.582 ± 0.088**	0.536 ± 0.077	0.883 ± 0.025	0.825 ± 0.042
mHC	0.834 ± 0.018	0.937 ± 0.052	0.461 ± 0.059	0.987 ± 0.012	**0.615 ± 0.053**	0.580 ± 0.048	0.940 ± 0.013	0.877 ± 0.015
mHC + RAG-LLM	0.868 ± 0.011	0.907 ± 0.046	0.610 ± 0.052	0.973 ± 0.015	**0.727 ± 0.034**	0.668 ± 0.033	0.946 ± 0.015	0.896 ± 0.011
**NC (mask ratio = 0.9)**								
Transformer	0.626 ± 0.036	0.594 ± 0.022	0.990 ± 0.009	0.193 ± 0.074	**0.743 ± 0.020**	0.303 ± 0.099	0.714 ± 0.019	0.714 ± 0.016
mHC	0.639 ± 0.012	0.602 ± 0.009	0.988 ± 0.011	0.223 ± 0.023	**0.748 ± 0.008**	0.338 ± 0.029	0.787 ± 0.054	0.771 ± 0.065
mHC + RAG-LLM	0.798 ± 0.016	0.828 ± 0.030	0.795 ± 0.027	0.800 ± 0.051	**0.810 ± 0.012**	0.595 ± 0.034	0.862 ± 0.026	0.852 ± 0.037
**MCI (mask ratio = 0.9)**								
Transformer	0.791 ± 0.017	0.243 ± 0.096	0.105 ± 0.041	0.928 ± 0.025	**0.142 ± 0.046**	0.051 ± 0.054	0.563 ± 0.015	0.225 ± 0.020
mHC	0.770 ± 0.013	0.172 ± 0.031	0.097 ± 0.019	0.905 ± 0.016	**0.124 ± 0.021**	0.004 ± 0.024	0.590 ± 0.035	0.218 ± 0.011
mHC + RAG-LLM	0.737 ± 0.039	0.314 ± 0.061	0.471 ± 0.068	0.790 ± 0.034	**0.377 ± 0.066**	0.226 ± 0.089	0.635 ± 0.045	0.274 ± 0.044
**DE (mask ratio = 0.9)**								
Transformer	0.722 ± 0.005	0.893 ± 0.137	0.047 ± 0.025	0.996 ± 0.005	**0.087 ± 0.044**	0.154 ± 0.034	0.595 ± 0.058	0.510 ± 0.067
mHC	0.722 ± 0.009	0.800 ± 0.400	0.042 ± 0.027	0.998 ± 0.004	**0.079 ± 0.051**	0.142 ± 0.103	0.751 ± 0.072	0.640 ± 0.074
mHC + RAG-LLM	0.814 ± 0.033	0.739 ± 0.095	0.563 ± 0.064	0.916 ± 0.033	**0.636 ± 0.063**	0.525 ± 0.088	0.821 ± 0.050	0.742 ± 0.069

Supplementary Table 5. Performance comparison of mHC, mHC + RAG-LLM, and Transformer baseline under progressive feature sparsity. performance for Global Label, Primary Label (subset1), MCI Subtypes Label (subset2), and Etiologies Label (subset3).

Comparison model	F1-macro	AUC-macro	AUPR-macro	subset1 F1	subset2 F1	subset3 F1
**mask ratio = 0**						
Transformer	0.277 ± 0.031	0.744 ± 0.036	0.302 ± 0.023	0.575 ± 0.023	0.044 ± 0.042	0.316 ± 0.028
mHC	0.287 ± 0.025	0.742 ± 0.029	0.297 ± 0.026	0.580 ± 0.038	0.064 ± 0.035	0.320 ± 0.022
mHC + RAG-LLM	0.338 ± 0.029	0.827 ± 0.014	0.390 ± 0.029	0.739 ± 0.045	0.086 ± 0.065	0.345 ± 0.031
**mask ratio = 0.3**						
Transformer	0.349 ± 0.024	0.831 ± 0.005	0.367 ± 0.026	0.748 ± 0.025	0.108 ± 0.036	0.346 ± 0.019
mHC	0.358 ± 0.031	0.819 ± 0.032	0.389 ± 0.018	0.767 ± 0.013	0.140 ± 0.041	0.339 ± 0.041
mHC + RAG-LLM	0.371 ± 0.023	0.822 ± 0.022	0.402 ± 0.011	0.774 ± 0.023	0.143 ± 0.064	0.361 ± 0.014
**mask ratio = 0.6**						
Transformer	0.287 ± 0.016	0.752 ± 0.021	0.315 ± 0.018	0.606 ± 0.036	0.069 ± 0.012	0.307 ± 0.025
mHC	0.290 ± 0.049	0.743 ± 0.021	0.336 ± 0.017	0.632 ± 0.019	0.111 ± 0.036	0.271 ± 0.088
mHC + RAG-LLM	0.322 ± 0.020	0.751 ± 0.028	0.346 ± 0.021	0.708 ± 0.025	0.106 ± 0.032	0.311 ± 0.031
**mask ratio = 0.9**						
Transformer	0.105 ± 0.040	0.583 ± 0.028	0.215 ± 0.030	0.323 ± 0.029	0.018 ± 0.024	0.043 ± 0.069
mHC	0.095 ± 0.010	0.594 ± 0.024	0.218 ± 0.018	0.317 ± 0.019	0.026 ± 0.016	0.049 ± 0.028
mHC + RAG-LLM	0.161 ± 0.020	0.665 ± 0.038	0.254 ± 0.018	0.608 ± 0.039	0.034 ± 0.017	0.061 ± 0.033

Supplementary Table 6. Summary of Literature Corpus for RAG-LLM. High-quality literature defined as publications after 2017 with >10 citations.

Literature Type	Alzheimer's Disease-Related	Cognitive Impairment-Related	Subtotal
High-Quality	3,357	9,855	13,212
General	13,385	27,406	40,791
Total	16,742	37,261	54,003

Supplementary Table 7. Demographic Characteristics of Participating Clinicians.

Category	Answer	Count (n)	Percentage (%)
Title	Chief Physician	160	40
	Resident Physician	160	40
	Attending Physician	80	20
Department	Psychiatry / Psychology	240	60
	Traditional Chinese Medicine	80	20
	Other (Unspecified)	80	20
Years of Experience	5 –10 years	160	40
	> 20 years	160	40
	< 5 years	80	20

Supplementary Table 8. Summary of clinicians' feedback.

Category	Representative Feedback
Treatment Recommendations	"Clarify that cholinesterase inhibitors represent one symptomatic treatment option, not the sole therapeutic choice."
	"Incorporate recommendations for disease-modifying therapy (DMT) in appropriate cases, especially if Alzheimer's disease (AD) is confirmed."
	"Emphasize early AD screening in patients with mild cognitive impairment (MCI) to facilitate timely DMT initiation."
	"The recommendation for SSRIs in depression should be expressed with more caution, avoiding absolute language."
Neuroimaging(MRI) Content	"MRI reports should include an assessment of cortical and hippocampal atrophy, not focus solely on cerebrovascular findings."
	"Highlight the urgency of conditions such as subdural or epidural hemorrhage more prominently."
Evidence Chain and Reasoning	"The evidence supporting an 'EXC' diagnosis based on "movement disorder, unspecified" is weak; the reasoning chain requires stronger support."
	"Information not present in the source data (e.g., a logical memory delay score of 2) was introduced in the report."
	"Inconsistency noted: the original MoCA score was presented as an MMSE score."
	"The report omitted mention of severe naming impairment, a critical clinical feature."
	"The reasoning process does not adequately incorporate the patient's chief complaint and disease course."
Clinical Recommendations	"The possibility of AD as a co-existing etiology should not be dismissed based solely on vascular factors; AD screening should be considered."
	"For patients diagnosed with AD, note that cholinesterase inhibitors are only one component of the therapeutic landscape."
Scale Interpretation	"In early-stage disease, the MoCA has higher sensitivity. When results from the MoCA and MMSE are discordant, the MoCA is often the preferred reference, warranting further AD-related biomarker testing."
Readability and Structure	"The report format was noted to be not optimally readable."
Positive Feedback	"The report was considered clear and easy to follow."
	"The logic was described as rigorous, with a relatively complete evidence chain and no over-inference."
	"The overall reasoning process was considered appropriate."
	"The report was noted to be relatively complete, with minimal discrepancies from the source data."
General Concerns	"There were instances of information without a clear source (e.g., specific functional details like financial management, appointment-keeping, and current events)."
	"A secondary diagnosis of AD was speculated without supporting biomarkers or clinical history."
	"Instances of information fabrication were noted (e.g., details regarding shopping and following current events)."

Supplementary Notes

Supplementary Note 1. Prompt for Cognitive Impairment Staging.

Similar Case Summary Prompt:

```
"""
As a medical expert, analyze the following similar cases that share contradictions with the current patient.
Provide a concise summary (max 300 words) of the diagnostic patterns observed in these cases.

Similar Case References:
{cases_block}

Patient Contradictions:
{patient_contradictions_block}

Please provide:
1. Common diagnostic patterns among cases with similar contradictions
2. How these contradictions were reconciled relative to the reported ground truths
3. Key insights or cautions for the current case
4. Explicitly list the case IDs referenced in your summary

Keep the summary focused and clinically relevant.
"""
```

Multimodal Diagnostic Reasoning Prompt:

```
"""
You are a senior neurologist conducting cognitive impairment diagnosis. Integrate multiple sources of information and provide a comprehensive diagnostic report in English.

### 1.Multimodal(mHC) prediction
{ml_reference}

### 2. STRICT Verification Protocol (Read Carefully)
**The AI prediction is highly reliable.** Do NOT change it unless you find a fatal error.

**Your Rule of Thumb:** "In dubio pro reo" (When in doubt, stick to the AI prediction).

**MODE A: Verification (If Anchor is NOT "NONE")**
1.   **Default Position**: You intend to **CONFIRM** the AI prediction.
2.   **Burden of Proof**: To override the AI, you must find **EXPLICIT, TEXTUAL EVIDENCE** that makes the prediction impossible.
       - *Example of Valid Override*: AI says "NC" (Normal), but text says "MoCA score: 12/30"
```

(Severe impairment).

  - *Example of INVALID Override*: AI says "MCI", text is vague about ADLs -> **KEEP MCI**. Do not assume impairment if not stated.

3. **Ambiguity Rule**: If the patient text is missing details (e.g., ADL status is unclear), **YOU MUST RETAIN THE AI PREDICTION**.

MODE B: Generation (If Anchor Hypothesis IS "NONE")

* **Directive**: Diagnose from scratch based solely on the patient data.
* **Process**: Analyze symptoms -> Assess ADLs -> Determine Label step-by-step.

3. Patient Data (Evidence)
Patient Description
{patient_text}

Similar Cases Summary
{similar_cases_summary}

Use these validation metrics as confidence context when balancing the AI prior against patient-specific evidence.

4. Classification Rules
Diagnostic Classification Tree
```
Cognitive Impairment Diagnosis
├── NC (Normal Control)
├── MCI (Mild Cognitive Impairment)
│   ├── MCI_A (Amnestic)
│   ├── MCI_AM (Amnestic Multi-domain)
│   ├── CI (pre-MCI)
│   ├── MCI_Na (Non-amnestic)
│   └── MCI_NaM (Non-amnestic Multi-domain)
└── DE (Dementia)
    ├── AD (Alzheimer's Disease)
    ├── LBD (Lewy Body)
    ├── VD (Vascular)
    ├── FTD (Frontotemporal)
    ├── EXC (movement disorder,reversible)
    ├── PSY (psychiatric)
    └── ODE (Other)
```
5. Analysis Checkpoints
Instruction:
- In **Verification Mode**: Use these checkpoints to *challenge* the prediction (look for

````
counter-evidence).
- In **Generation Mode**: Use these checkpoints to *derive* the correct label.

**Checkpoint A: The NC vs. Impairment Boundary**
*	*If Prediction is NC*: Does the patient have objective cognitive decline (low MoCA/MMSE) or confirmed biomarkers? (If yes -> Contradiction).
*	*If Prediction is MCI/DE*: Is the cognition objectively normal? (If yes -> Contradiction).

**Checkpoint B: The MCI vs. Dementia (DE) Boundary**
*	*Crucial Test*: **Activities of Daily Living (ADLs)**.
*	*If Prediction is MCI*: Are ADLs significantly impaired due to cognition? (If yes -> Override to DE).
*	*If Prediction is DE*: Are ADLs preserved/independent? (If yes -> Override to MCI).

**Checkpoint C: Subtype Consistency**
*	*If Prediction specifies a subtype (e.g., AD, LBD)*: Does the clinical text mention specific features (e.g., hallucinations for LBD, hippocampal atrophy for AD) that support or refute this?
*	*Mixed Etiology*: If the prediction misses a co-pathology (e.g., prediction is AD, but MRI shows significant vascular damage), add the secondary label.

**Labeling Rules:**
1. **Primary Label**: Must be one of [NC, MCI, DE].
2. **Secondary Labels**: Must be specific subtypes. Use 'NONE' if the primary label is NC.
3. **Override Rule**: You may only change the label if the clinical note contains factual evidence that makes the prior prediction impossible.
{critique_instruction}

### 6. Output Format (STRICT)
You must output a JSON object.
Before the JSON, you must include a `<thinking>` block to show your step-by-step logic.

Format structure:
<thinking>
1. Symptom Analysis: ...
2. ADL Assessment: ...
3. Consistency Check with ML ({ml_section}): ...
4. Final Conclusion Logic: ...
</thinking>

```json
{{
	"primary_label": "NC" | "MCI" | "DE",
	"secondary_labels": ["subtype1", "subtype2"] or ["NONE"],
	"mHC_prediction": {ml_reference},
````
````

```
        "confidence": 0-100 (Integer),
        "clinical_reasoning": "Concise summary of why this label was chosen..."
        "clinical_recommendations": "Recommended next steps or investigations..."
}}
"""
```

Supplementary Note 2. Sample Response for Cognitive Impairment Staging.

Sample Responses from Claude-Sonnet:

```
**Primary Label**: DE
**Secondary Labels**: AD

**Reasoning Process**:
1. **Data Quality Assessment**: The anthropometric measurements (weight 63.9 lbs, height 170.3 inches) are physiologically impossible for an adult human, and the Digit Span Backward data shows internal inconsistency (8 correct trials for length 7). Despite these data quality issues, sufficient clinical information remains for diagnostic assessment.
2. **Normal Control Exclusion**: The patient cannot be classified as NC due to significant functional impairment across multiple IADL domains (financial management, appointments, media comprehension, travel, shopping) and the presence of neuropsychiatric symptoms (nighttime behaviors, agitation/aggression, irritability).
3. **MCI vs. Dementia Distinction**: The extensive functional dependence in 5 IADL domains represents severe impairment that exceeds MCI criteria. While the MMSE score of 26 appears normal, this creates a classic screening-clinical reality disconnect pattern observed in the similar cases analysis. The neuropsychiatric symptoms (agitation, nighttime behaviors) further support a dementia diagnosis over MCI.
4. **Dementia Subtype Analysis**:
   - **AD Evidence**: The combination of memory-related functional deficits (appointments, financial management), behavioral symptoms (agitation, nighttime behaviors), and the multimodal AI prediction strongly supports Alzheimer's disease
   - **LBD Evidence**: No clear visuospatial deficits, REM sleep behavior disorder, or motor symptoms documented
   - **VD Evidence**: Hypertension present (138/68) but no focal neurological signs or clear vascular imaging findings
   - **FTD Evidence**: No prominent language, behavioral disinhibition, or executive dysfunction patterns typical of FTD
5. **Integration with AI Prediction**: The multimodal AI prediction of "DE" aligns with clinical assessment. Given the model's 92% recall for dementia detection and 70% precision, this strengthens confidence in the dementia diagnosis.

**Clinical Confidence & Recommendations**:
- **Confidence Level**: Moderate-High (75%) for dementia diagnosis, Moderate (65%) for AD subtype
- **Key Uncertainties**: The normal MMSE score creates diagnostic tension, though similar contradictory cases in the reference set ultimately received dementia diagnoses when clinicians weighted functional decline over screening scores
- **Recommended Next Steps**:
  1. Comprehensive neuropsychological testing to identify specific cognitive domains affected
```

2. Repeat anthropometric measurements with calibrated equipment
3. Consider CSF biomarkers or amyloid PET imaging for AD confirmation
4. Detailed medication review and metabolic workup to exclude reversible causes
5. Caregiver interview to validate functional assessment and obtain collateral history
6. Sleep study evaluation given prominent nighttime behavioral symptoms

The diagnosis prioritizes the substantial functional impairment and behavioral symptoms over the contradictory normal MMSE score, consistent with clinical judgment patterns observed in similar complex cases.

Sample Responses from DeepSeek-R1:

Primary Label: NC
Secondary Labels: NONE

Reasoning Process
1. **Cognitive Performance Validation**:
   - MoCA score is 26 (corrected), within normal limits (≥26). All domains (visuospatial/executive, orientation, memory) are intact.
   - Logical Memory: Immediate recall (18 units) and delayed recall (14 units) are strong, exceeding typical impairment thresholds.
   - MMSE confirms "perfect cognitive function."
   - No deficits in attention, language, or executive function noted.
2. **Functional Independence**:
   - Fully independent in all IADLs (financial tasks, cooking, medication management, travel, shopping).
   - No evidence of functional decline impacting daily life, a core criterion for MCI/DE.
3. **Behavioral Symptoms vs. Cognitive Status**:
   - Agitation, disinhibition, irritability, and appetite issues are present but **do not correlate** with cognitive/functional impairment.
   - GDS=0 (no depression) contradicts subjective negative affect, suggesting possible:
     - Underlying psychiatric disorder (e.g., adjustment/anxiety disorder).
     - Limitations of GDS in capturing atypical behavioral presentations.
   - *Key Insight*: Behavioral changes without cognitive decline are atypical for neurodegenerative etiologies but align with psychiatric conditions.
4. **Neuroimaging & Medical History**:
   - UniBrain MRI: Normal, ruling out structural causes (e.g., atrophy, vascular lesions).
   - Absence of vascular risk factors (normal BP, no diabetes/stroke history) and focal neurological signs.
5. **AI Prediction Alignment**:
   - Multimodal AI predicts NC (accuracy 73.94%), and clinical evidence aligns without contradiction. Behavioral anomalies are not cognitive indicators.
6. **Contradiction Resolution**:
   - Similar cases (NACC275936) show NC diagnosis despite behavioral symptoms, emphasizing functional/cognitive preservation as the diagnostic anchor.

   - GDS=0 may reflect anosognosia or scale limitations but does not override objective cognitive normality.

Clinical Confidence & Recommendations
- **Confidence**: High (85%) for NC.
  - *Uncertainties*: Behavioral symptoms unexplained; GDS discordance.
  - *Not neurodegenerative*: No progressive cognitive/functional decline.
- **Recommendations**:
  1. **Psychiatric Evaluation**: Rule out anxiety, adjustment disorders, or atypical depression using tools like NPI-Q.
  2. **Medical Workup**: Thyroid function, B12/folate, electrolytes to exclude organic causes of behavioral changes.
  3. **Anthropometric Verification**: Recheck height (invalid value: 183.2 inches).
  4. **Monitoring**: Annual cognitive screening; track behavioral symptoms for conversion (low risk based on current data).
  5. **Non-Pharmacological Intervention**: Behavioral therapy for symptom management.

Key Insight: Behavioral symptoms in cognitively intact patients warrant psychiatric—not neurodegenerative—evaluation. GDS limitations necessitate nuanced clinical assessment.

Sample Responses from GPT-5:

Primary Label: MCI
Secondary Labels: MCI_NaM

Reasoning Process:
1) Step 1 – NC vs Impairment
- Global screens: MMSE 26 is borderline; MoCA subscores available are intact for visuospatial/executive items listed and full orientation intact, but the overall profile is not clearly normal.
- Neuropsychology shows clear abnormalities: Trail Making Test B = 282 sec (severely impaired), Trail A = 40 sec (borderline), category fluency low (Vegetables 6; Animals 12), with normal digit span. These indicate executive and semantic retrieval deficits.
- Functional impact: Needs assistance with finances, remembering appointments/medications, assembling tax records—executive and prospective memory-dependent IADLs—despite otherwise broad independence.
- Conclusion: Not Normal Control.

2) Step 2 – MCI vs Dementia
- Functional status: Independent in most IADLs (cooking, shopping, travel, hobbies, reading/TV attention, current events). Selective dependence in higher-order, executive-heavy tasks suggests mild functional compromise, not pervasive loss of independence typical of dementia.
- Cognitive severity: Severe executive test impairment with otherwise preserved orientation and visuospatial MoCA items; Logical Memory immediate 14 and delayed 15 (delayed > immediate is atypical and likely a scoring/attention/practice artifact; does not support a frank amnestic syndrome).

- Therefore, overall pattern fits Mild Cognitive Impairment rather than Dementia.
- This aligns with the Multimodal AI prior (MCI), which we weight strongly given no definitive clinical contradiction.

3) Step 3 – MCI Subtype
- Amnestic vs non-amnestic: Memory is not convincingly impaired; narrative delayed recall equals/exceeds immediate and orientation is intact. Deficits center on executive control and lexical-semantic retrieval (set-shifting, processing speed, category fluency). Thus non-amnestic.
- Single vs multi-domain: Impairments encompass multiple non-memory domains—set-shifting/processing speed (TMT A/B), verbal fluency (semantic categories), with relative preservation of attention span and visuospatial construction. This constitutes non-amnestic multi-domain MCI (MCI_NaM).

4) Reconciling contradictions and anomalies
- Depression/dysphoria vs GDS=0: Discordant signals suggest under-reporting on GDS or fluctuating mood/irritability; mood symptoms could exacerbate executive inefficiency but do not explain the magnitude of Trails B impairment alone. Collateral and alternative mood scales needed.
- Delayed > immediate recall: Most likely scoring or attention fluctuation; treat as artifact pending corroboration with list learning (e.g., HVLT-R/CVLT) and recognition measures.
- ADL discrepancies: Selective dependence in finance/medication aligns with executive MCI, while preserved travel/cooking supports non-demented status.
- MRI flags (epidural/subdural hemorrhage, meningioma, hemangioma): If accurate and current, these are potentially consequential structural pathologies. Coexistence with “normal” suggests either historical/incidental findings or report aggregation artifact. Any active subdural/epidural collection or mass effect could drive executive deficits and mandates urgent radiologic clarification.

5) Dementia subtype screen (if we were to consider DE)
- AD: Lacks clear amnestic pattern or documented medial temporal atrophy; not favored.
- LBD: No parkinsonism, hallucinations, or visuospatial deficits; unlikely.
- Vascular: No vascular risk factors reported; Trails pattern could fit subcortical/vascular executive dysfunction, but imaging evidence of small vessel disease not provided.
- FTD: Behavioral changes limited to irritability; no disinhibition/apathy/loss of empathy/compulsions; language preserved. Not supported.

Clinical Confidence & Recommendations:
- Confidence: Moderate that diagnosis is MCI_NaM, anchored to AI prior and supported by prominent executive dysfunction with selective IADL impact. Uncertainties stem from discordant mood measures, the delayed>immediate memory anomaly, and ambiguous MRI report listing serious lesions alongside “normal.”
- Immediate actions:
  1) Neuroimaging clarification: Obtain and review actual MRI report and images with neuroradiology. Confirm presence, chronicity, size, and mass effect of any epidural/subdural hemorrhage and meningioma/hemangioma. If any acute/subacute collection or significant mass effect is present, expedite neurosurgical consultation.

2) Vascular screen: Basic labs and risk assessment (BP monitoring, fasting lipids, HbA1c, B12, TSH, folate). Consider sleep apnea screen if snoring/daytime sleepiness or bradycardia-related concerns.

3) Mood/behavior evaluation: Repeat mood assessment with PHQ-9, NPI-Q (caregiver), and clinician interview; assess for apathy vs depression. Treat mood symptoms if present; re-evaluate cognition after stabilization.

4) Comprehensive neuropsychological testing: Include list learning (HVLT-R/CVLT), Stroop, Wisconsin/modified card sorting, Trail B again, phonemic and semantic fluency, Boston Naming, design fluency. This will better define domains and rule out effort/scoring artifacts.

5) Functional and safety: Collateral IADL inventory (e.g., FAQ). Consider performance-based executive function measures (e.g., Pillbox Task, Weekly Calendar Planning Test). Implement practical supports now for finances and medications (automatic bill pay, pill organizer, reminders).

6) Reversible contributors: Review for anticholinergic or sedating agents (none reported), alcohol use, sleep, stress, and sensory deficits (vision/hearing).

7) Follow-up: Repeat cognitive testing in 6–12 months or sooner if decline; adjust diagnosis if functional losses progress.

8) If structural lesions are chronic/incidental and deficits persist, consider etiologic workup for frontal/executive MCI including small vessel disease markers on MRI (FLAIR white matter hyperintensities, microbleeds on SWI), and, if AD concern emerges, consider biomarkers (CSF Aβ42/40, p-tau, or amyloid/tau PET) guided by clinical evolution.

Overall, current evidence supports Mild Cognitive Impairment, non-amnestic multi-domain, with a frontal/executive predominance and unresolved imaging concerns that require prompt clarification.

Supplementary Note 3. Label Correction:

Correct Label Sample (True Label: MCI)

```json
{
    "primary_label": "MCI",
    "secondary_labels": ["MCI_AM"],
    "mHC_prediction": "NC",
    "confidence": 78,
    "clinical_reasoning": "Override of NC prediction justified by explicit objective evidence: MoCA 18/30 (significantly impaired, 8 points below cutoff of 26), documented visuospatial/executive deficits (incorrect cube drawing, trails), and disorientation to place. Memory performance also appears reduced (delayed recall 6.0). Despite these cognitive deficits, ADLs remain fully preserved (independent in financial management, cooking, shopping, medication management, travel), which definitively places this patient in MCI rather than dementia. The multi-domain involvement (memory + executive + orientation) with amnestic features supports MCI_AM subtype. The MMSE-MoCA discrepancy (26 vs 18) reflects MoCA's greater sensitivity to executive dysfunction. MRI focal ischemia noted but Hachinski score minimal, suggesting vascular pathology is not primary driver.",
    "clinical_recommendations": "1) Urgent cardiovascular evaluation for undiagnosed hypertension (BP 173/105); 2) Repeat neuropsychological testing in 6-12 months to monitor progression; 3) Neurosurgical consultation for incidental meningioma; 4) Consider amyloid PET or CSF biomarkers given family history and age; 5) Screen for depression given somatic complaints; 6) Optimize vascular risk factors; 7) Cognitive rehabilitation focusing on executive function strategies."
}
```

```
<thinking>
```

1. Symptom Analysis:

- MoCA score: 18/30 (impaired, cutoff typically 26)
- MMSE score: 26/30 (borderline normal)
- Visuospatial/Executive deficits: Cube drawing incorrect, Trails incorrect
- Orientation deficit: Disoriented to place
- Logical Memory: Immediate recall 8.0, Delayed recall 6.0 (need norms but appears low)
- Somatic complaints present
- No focal neurological signs
- MRI shows focal ischemia and meningioma

2. ADL Assessment:

- CRITICAL: Patient is "Fully independent in all ADLs/IADLs (financial management, cooking, meal preparation, travel, shopping, medication management)"
- No urinary/bowel incontinence
- Lives independently in retirement community

3. Consistency Check with ML Prediction (NC):
- However, there is EXPLICIT evidence of cognitive impairment:
  * MoCA 18/30 is significantly impaired (8 points below cutoff)
  * Visuospatial/executive deficits documented
  * Disorientation to place
  * Memory scores appear reduced
- This constitutes a VALID OVERRIDE scenario: AI says NC but objective cognitive testing shows clear impairment

4. Final Conclusion Logic:
- The patient has objective cognitive impairment (MoCA 18, executive deficits, orientation issues)
- However, ADLs are FULLY PRESERVED
- Per Checkpoint B: If ADLs are preserved/independent, this supports MCI over DE
- The cognitive profile shows:
  * Memory involvement (delayed recall 6.0)
  * Executive dysfunction (trails, cube)
  * Orientation deficit
- This suggests multi-domain involvement with amnestic features → MCI_AM (Amnestic Multi-domain)
- The MRI findings (focal ischemia) may contribute but Hachinski score is minimal for vascular dementia
- Family history of maternal cognitive impairment is a risk factor

Override Justification: The MoCA score of 18/30 is explicit, objective evidence of cognitive impairment that makes the NC prediction impossible. This is a valid override per the protocol.

Label Correction: Incorrect Label Sample (True Label: DE)

```json
{
	"primary_label": "MCI",
	"secondary_labels": ["MCI_AM"],
	"mHC_prediction": "DE",
	"confidence": 72,
	"clinical_reasoning": "Despite severe cognitive impairment on testing (MMSE=20, Trail Making A=150 sec), the patient maintains explicit independence in ALL instrumental ADLs including financial management, cooking, travel, and shopping. Per diagnostic criteria, dementia requires functional impairment due to cognitive decline. The preserved ADL function constitutes explicit textual evidence warranting override from DE to MCI per Checkpoint B. The cognitive profile shows both memory deficits (story recall, logical memory) and executive dysfunction (severely impaired Trails A, reduced digit span backward), consistent with amnestic multi-domain MCI. Brain atrophy on MRI supports underlying neurodegenerative process. The discrepancy between test performance and functional status may reflect high cognitive reserve (17 years education) or informant reporting bias.",
	"clinical_recommendations": "1) Repeat cognitive testing in 6-12 months to monitor progression; 2) Obtain collateral ADL assessment from family member to verify functional status; 3) Neurology follow-up for meningioma and hemangioma monitoring; 4) Consider amyloid PET or CSF biomarkers to evaluate
```

Alzheimer's pathology; 5) Address hypertension (160/84) as vascular risk factor; 6) Behavioral management for anxiety and disinhibition; 7) Occupational therapy evaluation for subtle functional deficits."
}
```

1. Symptom Analysis:
- MMSE = 20/30 indicates moderate-severe cognitive impairment
- Trail Making A = 150 seconds is severely impaired (normal <78 sec for age)
- Digit Span Forward 4/4, Backward 1/2 shows attention/working memory deficits
- Story recall = 9, Logical Memory IIA delayed = 8 (need norms but suggests memory involvement)
- Category fluency: Vegetables 9, Animals 10 - borderline low
- Behavioral symptoms: Anxiety, disinhibition present
- MRI: Brain atrophy, hemangioma, meningioma

2. ADL Assessment:
- Patient is described as INDEPENDENT in ALL instrumental ADLs: financial management, cooking, meal prep, hobbies, appointment recall, travel, shopping, current events tracking
- No incontinence
- This is a critical finding - preserved ADLs typically indicate MCI rather than dementia

3. Consistency Check with ML Prediction (DE):
- The mHC prediction is DE (Dementia)
- However, the clinical data shows PRESERVED instrumental ADLs
- The key diagnostic boundary between MCI and DE is ADL impairment
- MMSE of 20 is consistent with dementia-level cognitive impairment
- BUT: The patient maintains independence in complex activities (financial management, travel, shopping)

4. Critical Analysis:
- This is a contradiction case: severe cognitive test scores (MMSE=20, Trails A=150) but preserved functional independence
- Per Checkpoint B: "If Prediction is DE: Are ADLs preserved/independent? (If yes -> Override to MCI)"
- The clinical evidence explicitly states independence in ALL instrumental ADLs
- This is EXPLICIT TEXTUAL EVIDENCE that contradicts the DE prediction

5. Final Conclusion Logic:
- Despite severe cognitive test impairment, the preserved ADL function is explicit evidence
- Per the verification protocol, ADL preservation with cognitive impairment = MCI, not DE
- The discrepancy between cognitive scores and functional status is flagged as an anomaly
- However, the diagnostic criteria for dementia REQUIRE functional impairment
- This meets criteria for MCI (cognitive impairment without significant functional decline)
```

- Subtype: Given memory involvement (story recall, logical memory) plus executive dysfunction (Trails A, digit span backward) = Multi-domain, likely amnestic multi-domain (MCI_AM)
- Brain atrophy on MRI supports neurodegenerative process
- Meningioma and hemangioma may be incidental but warrant monitoring

Override Decision: The explicit statement of preserved ADLs is valid evidence to override DE to MCI per Checkpoint B rules.
</thinking>
